# Biomolecular condensates sustain pH gradients at equilibrium through charge neutralization

**DOI:** 10.1038/s41557-025-02039-9

**Published:** 2026-01-29

**Authors:** Hannes Ausserwöger, Rob Scrutton, Charlotte M. Fischer, Tomas Sneideris, Daoyuan Qian, Ella de Csilléry, Ieva Baronaite, Kadi L. Saar, Alan Z. Białek, Marc Oeller, Georg Krainer, Titus M. Franzmann, Sina Wittmann, Juan M. Iglesias-Artola, Gaetano Invernizzi, Anthony A. Hyman, Simon Alberti, Nikolai Lorenzen, Tuomas P. J. Knowles

**Affiliations:** 1 Centre for Misfolding Diseases, Yusuf Hamied Department of Chemistry, University of Cambridge, Cambridge, UK; 2 Proteomics and Signal Transduction, Max Planck Institute of Biochemistry, Martinsried, Germany; 3 Biophysics, Institute of Molecular Biosciences (IMB), NAWI Graz, University of Graz, Graz, Austria; 4 BioTechMed-Graz, Graz, Austria; 5 Field of Excellence BioHealth, University of Graz, Graz, Austria; 6 Biotechnology Center (BIOTEC), Center for Molecular and Cellular Bioengineering (CMCB), Technische Universität Dresden, Dresden, Germany; 7 Institute of Molecular Biology (IMB), Mainz, Germany; 8 Max Planck Institute of Cell Biology and Genetics (MPI-CBG), Dresden, Germany; 9 Therapeutics Discovery, Novo Nordisk A/S, Måløv, Denmark

**Keywords:** Biophysics, Physical chemistry, Proteins

## Abstract

Electrochemical gradients are essential to the functioning of cells and form across membranes using active transporters. Here we show in contrast that condensed biomolecular systems—often termed condensates—sustain pH gradients without any external energy input. By studying individual condensates on the micrometre scale using a microdroplet platform, we reveal dense-phase pH shifts towards conditions of minimal electrostatic repulsion. We demonstrate that protein condensates can drive substantial alkaline and acidic gradients, which are compositionally tunable and can extend to complex architectures sustaining multiple unique pH conditions simultaneously. Through in silico characterization of human proteomic condensate networks, we further highlight potential wide-ranging electrochemical properties emerging from condensation in nature, while correlating intracellular condensate pH gradients with complex biomolecular composition. Together, the emergent nature of condensation shapes distinct pH microenvironments, thereby creating a regulatory mechanism to modulate biochemical activity in living and artificial systems.

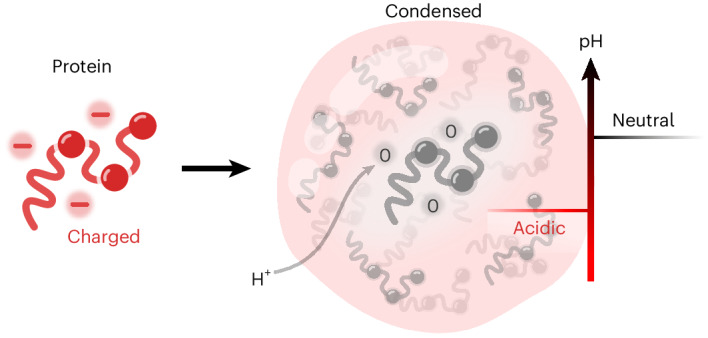

## Abstract (web summary)

pH is a critical regulator of (bio)chemical processes and therefore tightly regulated in nature. Now, proteins have been shown to possess the functionality to drive pH gradients without requiring energy input or membrane enclosure but through condensation. Protein condensates can drive unique pH gradients that modulate biochemical activity in both living and artificial systems.

## Main

The proton (H^+^) concentration, typically represented as the pH, is a uniquely important environmental factor in chemistry and biology. It affects all fundamental processes underlying viability and functioning of cells, including electrochemical potential gradients^1^, chemical reaction rates^2^ and macromolecular conformation and assembly^3,4^. Proteins’ function, for example, is highly susceptible to varying pH conditions owing to shifting protonation states and associated changes in physico-chemical properties. As a consequence, pH is tightly regulated within the intracellular environment, with deviations from the cytosolic pH previously only found in membrane-bound organelles^5,6^. Here, through energy input using active transporters, the organellar pH is commonly regulated to optimize for specific reactions and functional processes^7–12^. Precise control over the intracellular pH is further aided by a high buffering capacity of the cytosol. The cytosolic pH regulation primarily originates from smaller ionic species such as phosphates and carbonates^5,6^, with protein-associated side groups only accounting for about 1% of the buffering^13^.

The low-protein-concentration picture can be drastically altered as a result of biomolecular phase separation where protein-rich condensed phases, often referred to as condensates, are formed at equilibrium. These condensates are believed to play critical roles in cellular organization and pathology^14–16^. The underlying collective interactions shape unique chemical microenvironments with distinct material properties or differential molecular partitioning^17^, including formation of elemental ion gradients^18,19^. Reports even suggest that condensates could drive pH gradients intracellularly^20–25^. Hence, we sought a generalizable understanding of how pH influences the collective assembly of biomolecules as well as if condensed phases can counteract external pH conditions and establish pH gradients.

## Results

### Mapping pH-responsive phase boundaries in a continuous manner

**Fig. 1:**
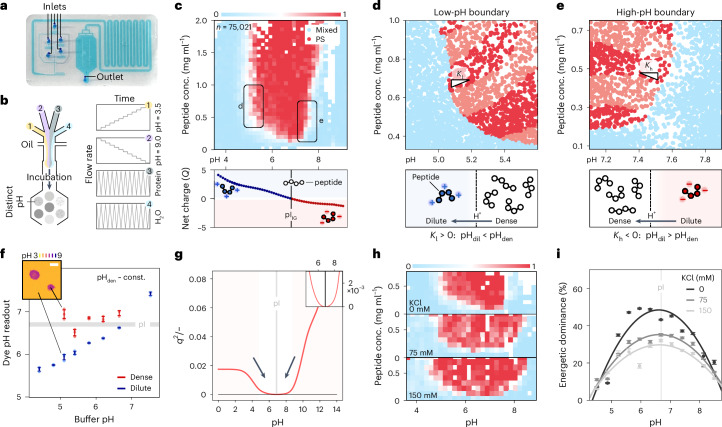
Deciphering pH-responsive macromolecular phase behaviour. **a**, Image of the microfluidic chip, filled with a coloured solution to highlight the device features, used to generate continuous pH phase diagrams. **b**, Schematic of the chip design and flow profiles used to generate continuous pH phase boundaries. **c**, pH-responsive phase boundary of an insulin variant peptide (insulin glargine), correlated with the molecular net charge (*Q*). Data are binned into a grid map to evaluate the local fraction of phase-separated conditions (*F*_PS_ = 1, red; *F*_PS_ = 0.5, white; *F*_PS_ = 0, blue) as determined by averaging (*n*_tot_ = 75,021). Data represent an individual repeat where the effect of sampling and repeat reproducibility have been tested further in Extended Data Fig. 2. **d**,**e**, Visualization of the dilute-phase contour gradients (*K*) at the low-pH (**d**) and high-pH (**e**) phase boundary. Dilute-phase contours are shown using red shading of phase-separated points as generated by sectioning for the dilute-phase concentration obtained in each individual microdroplet^18,31,33^. Lower panels: schematic representations visualizing the proton partitioning as inferred from the reduced tie-line gradients. **f**, Dense- and dilute-phase pH readouts of insulin glargine samples at varying pH conditions using the ratiometric pH-sensing dye SNARF-4F. Inset: representative image colour-coded for the pH readout. Scale bar, 10 μm. Data are presented as mean ± s.d. from *n* = 3 technical replicates. **g**, Change in the square of the sequence charge density (*q*) used as a proxy for the repulsion free energy with varying pH for insulin glargine. This showcases a minimum close to the pI as reflected by the directional arrows. **h**, Effect of the addition of KCl on the pH-responsive phase boundary of the insulin glargine (*n*_75mM_ = 12,623, *n*_150mM_ = 9,969). **i**, Quantification of the protein dominance with varying pH and salt concentrations for the insulin glargine. Data are presented as mean ± s.d.; parameter errors are estimated by repeated perturbation on fitting data and quantifying the spread of best-fit parameters (Extended Data Fig. 2). conc., concentration; const., constant. Source data

**Extended Data Fig. 1:**
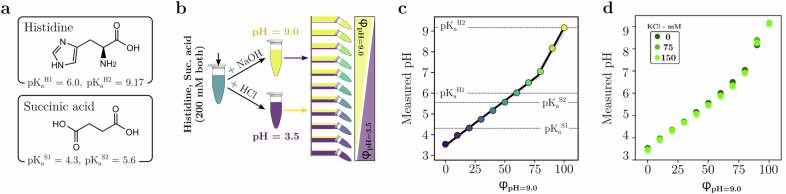
Histidine and succinic acid linear volume fraction pH buffering system. (**a**) Molecular structure of histidine and succinic acid including individual relevant pK_a_s. (**b**) Mixing scheme for generating the buffering stocks by adjusting the pH of mixed histidine and succinic acid stocks. (**c**) Correlation between the measured pH and the volume fraction of pH = 9 H/S buffer stock. (**d**) Salt dependence of the Histidine / Succinic acid buffer system. Source data

**Extended Data Fig. 2:**
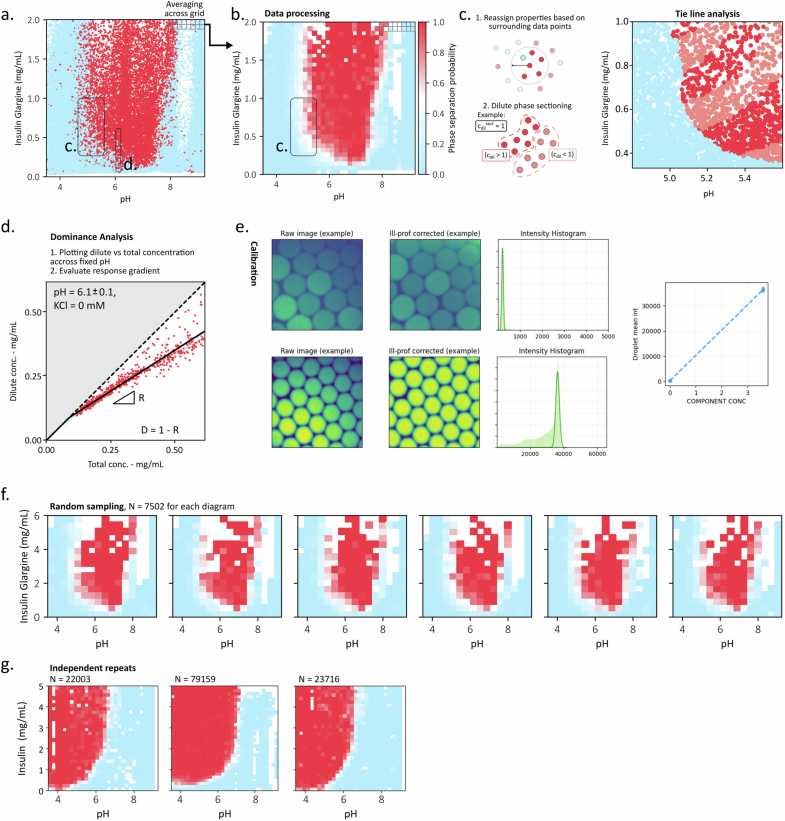
Data processing of microdroplet phase boundary mapping approach. (**a**) Raw data obtained from droplet-based phase boundary mapping. The density of data points (N = 75021) entails that simple scatter plotting is not visually representative of the results obtained given randomized overlapping of individual data points. (**b**) Grid-based averaging then allows to obtain representative visualisation of the data by considering the properties of all data points within a defined bin. Bin sizes were chosen based on established literature algorithms^3,4^. (**c**) Schematic explanation of the visualisation of dilute phase contours. Raw data are first averaged based on neighbouring data points in a defined radius (typically 3% of the axis scale) to generate a representative readout from the large data density. Dilute phase contours are then obtained by repeated sectioning for threshold dilute phase values. (**d**) Dominance analysis is then executed by evaluating the change in dilute phase concentration with varying total protein concentrations. Change in c_dil_ of insulin glargine with changing total concentration at a fixed pH and salt concentration (see marking box in a.). R - dilute phase response gradient. (**e**) Calibration and correction in droplet microfluidic mapping. Images of microdroplets containing one of the stock solutions only are first corrected for the Illumination profile (see Raw image vs Ill-pof corrected). Intensity histograms are then fitted to generate concentration calibrations enabling back calculation of the concentration in each individual droplet. This process is subsequently repeated for all fluorescently labelled solute streams. (**f**) Testing of reproducibility by random sampling of only 10% of the total data points. (**g**) Independent repeats of Insulin phase separation at varying pH highlight reproducibility of droplet-microfluidic approach. Source data

**Extended Data Fig. 3:**
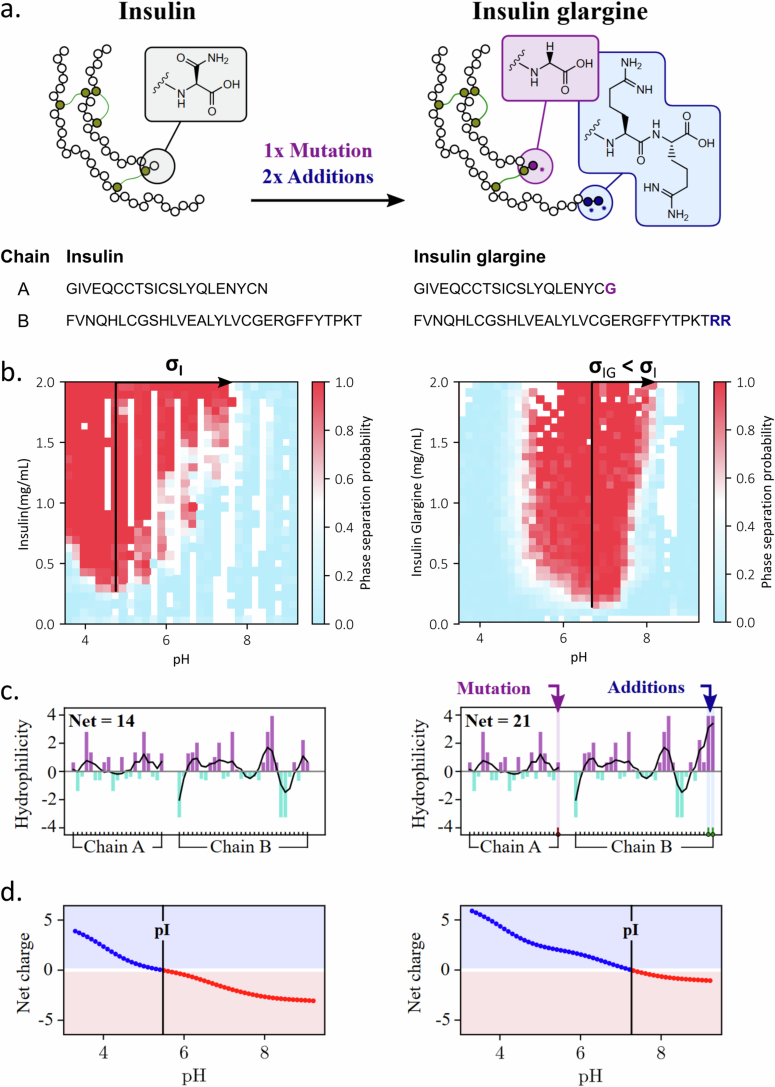
Sequence and phase behavior comparison of insulin and insulin glargine. (**a**) Schematic representation and summary of the sequence changes from insulin-to-insulin glargine. (**b**) pH-responsive phase boundaries for both peptides including indication of the boundary half-width. (**c**) Sequence hydrophobicity profiles for insulin and insulin glargine. (**d**) Sequence net charge profiles for insulin and insulin glargine. Source data

To quantify pH-responsive phase behaviour, we used a combinatorial droplet microfluidic platform, enabling the generation of large numbers of water-in-oil droplets with distinct chemical microenvironments^26^ (Fig. 1a,b). Continuous variation of the pH in droplets was achieved by integrating a histidine and succinic acid buffer system (H/S buffer), capable of regulating pH between 3.5 and 9 (Methods and Extended Data Fig. 1). Each droplet was then subjected to image analysis to characterize the phase state and subsequently processed to map the local phase behaviour (Fig. 1c and Extended Data Fig. 2). We first selected insulin and its clinically relevant variant, insulin glargine, because of their well-characterized pH-sensitive self-assembly^27–29^ and availability as model systems, enabling a direct assessment of how subtle sequence modifications influence phase separation across varying pH conditions. Application of this platform to insulin glargine (modified insulin variant; Extended Data Fig. 3) yielded a continuous map of over 75,000 data points of the pH-responsive phase boundary.

**Extended Data Fig. 4:**
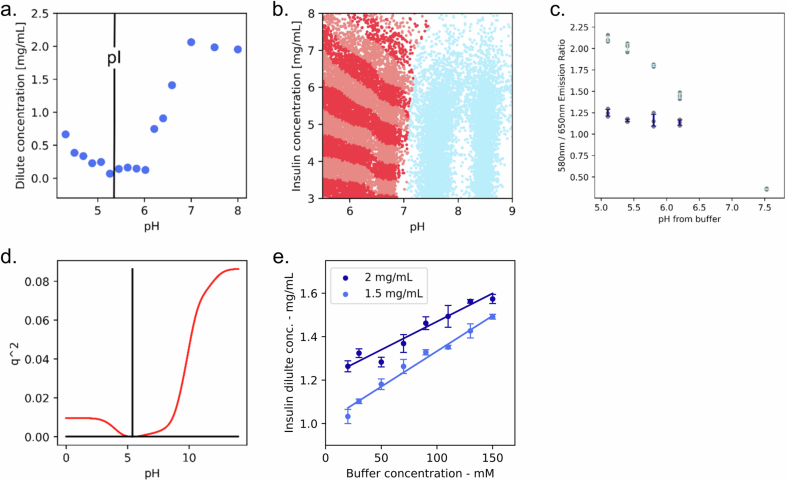
pH and buffer response of insulin phase separation. (**a**) Change in the insulin dilute phase concentration with varying pH using the histidine and succinic acid buffer system at 2 mg/mL total insulin concentration and constant H/S buffer concentration of 100 mM. The dilute phase concentration is minimized around the isoelectric point indicating that most protein is being converted into the dense phase around the pI. (**b**) Visualisation of the 2D reduced tie line gradients for insulin. Reduced tie line gradients are displayed using red shaded bands as generated by sectioning for the dilute phase concentration obtained in each individual microdroplet. (**c**) SNARF-4F readouts for insulin phase separation at varying pH. Emission maxima readout for both insulin dense (dark blue) and dilute phase (light blue) at varying start pH conditions, highlighting a large variation in the dilute phase but constant dense phase ratio. Data are presented as mean ± SD from n > 3 repeats. (**d**) pH dependent q2 profile of unmodified insulin. Approximation of the pH dependent repulsion free energy exerted by insulin in the condensed phase using the sequence charge density (q). (**e**) Effect of buffer strength on insulin phase separation. Insulin dilute phase concentration change with varying H/S buffer concentration at fixed pH = 6.4 at a total insulin concentration of 2 and 1.5 mg/mL. Data are represented as the mean ± SD from n > 3 repeats. Source data

Phase separation of insulin glargine was only observed close to the isoelectric point (pI, condition of overall charge neutrality) at 6.8, highlighting the importance of the sequence charge (*Q*). The data suggest that molecular charge neutrality is a requirement for associative interactions to become favourable. Insulin (pI_Ins_ = 5.4; glargine variant, N21G mutation, 2× R additions in the β-chain) shows a similar correlation between the phase boundary and the sequence charge, where in both cases the minimum in the critical concentration coincides with the pI (Extended Data Fig. 3). Accordingly, the dilute-phase concentration (*c*_dil_) also showed a minimum around the pI, meaning that phase separation propensity attains a maximum at the pI (Extended Data Fig. 4a).

A decrease in the width of the pH phase boundary of insulin glargine compared with unmodified insulin (Extended Data Fig. 3) coincides with an increase in hydrophilicity (*H*_pH=7, I_ = 14, *H*_pH=7, IG_ = 21; Kyte–Doolittle scale^30^). Addition of two charged arginine residues makes insulin glargine more hydrophilic, indicating a trade-off between intermolecular charge repulsion and attractive van der Waals interactions. It is worth noting that the phase boundary is not perfectly symmetrical, suggesting higher-order effects such as the individual ionization changes of specific amino acids.

### Polypeptide chains buffer pH towards charge-neutral conditions

Next, we set out to characterize the H^+^ partitioning between the dense and dilute phase by applying a previously published approach based on quantifying the peptide dilute-phase concentrations (*c*_dil_)^18,31,32^. In an exact two-component system, contours of constant *c*_dil_ coincide with tie-lines, which describe the demixing process by connecting the dilute- and dense-phase composition^18,33^. We mapped these contours by sectioning the dilute-phase concentration conditions in set thresholds of *a* < *c*_dil_ < *b* (Fig. 1d,e; light and dark red shaded contours; see Extended Data Fig. 2 for data processing). Other species, however, affect the slope of mentioned contours, referred to here as *K*, therefore, only providing a lower bound for a tie-line component ratio^31^. This distortion is dependent on the shape of the phase boundary, with minimal effect where the phase boundary is parallel to the peptide concentration axis such as at the high and low critical pH conditions observed here^31^.

When triggering insulin glargine phase separation at low pH (~5), the dilute-phase contour shows a positive gradient (Fig. 1d, *K*_l_ > 0), indicating that the pH in the dilute (pH_dil_) and dense phase (pH_den_) are not equivalent. Specifically, this suggests that pH_den_ > pH_dil_, that is, protons are being excluded from the dense-phase shifting pH closer to the pI at 6.8. At the high-pH phase boundary (pH ~8), protons partition into the dense phase (*K*_h_ < 0), also bringing the dense-phase pH closer to the pI (Fig. 1e). Hence, independent of the starting pH, the dense-phase pH shifts towards the pI, minimizing the molecular charge. A similar behaviour is observed for insulin at the high-pH boundary where *K*_pH_ < 0 (Extended Data Fig. 4b). The low-pH phase boundary could not be resolved with the available buffer system.

**Extended Data Fig. 5:**
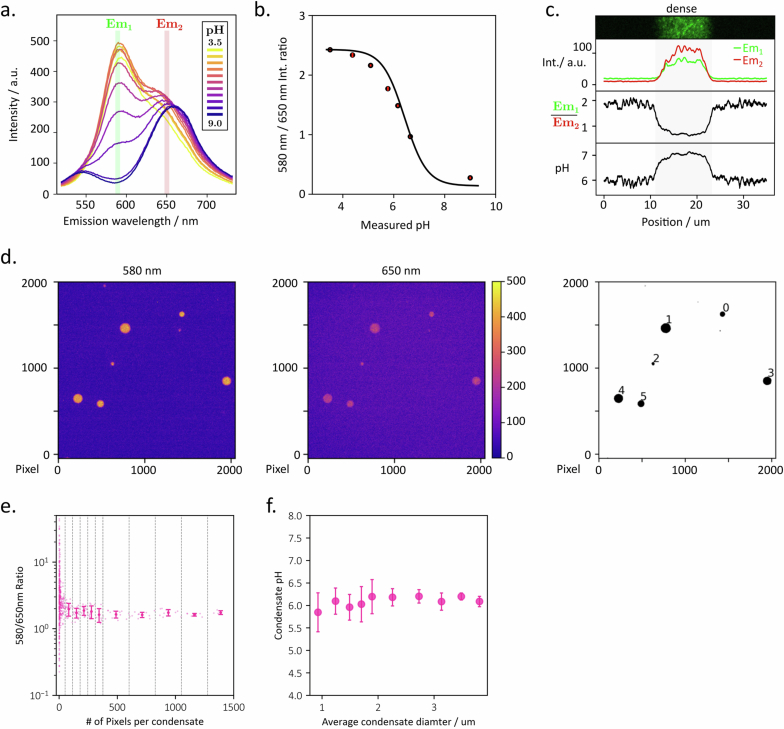
Ratiometric pH-sensing dye readout approach. (**a**) pH dependent emission spectrum shift. SNARF-4F dye emission spectrum post excitation at 488 nm at varying pH, highlighting two emission maxima at around 580 and 650 nm. (**b**) pH readout calibration of SNARF-4F. Change in SNARF-4F intensity ratio of the 580 nm and 650 nm emission maximum as obtained by confocal imaging of SNARF-4F at 20 uM in H/S buffer at varying pH. (**c**) Spatially resolved SNARF-4F based pH readout. Intensity at the 580 nm (Em_1_) and 650 nm (EM_2_) emission maxima, emission maxima ratio and pH conversion of SNARF-4F readouts in a spatially resolved manner between dense and dilute phase for insulin glargine at a starting pH of ~ 6 using H/S buffer. (**d**) Exemplary condensate detection mask from SNARF-4f readouts. Confocal image of PGL3 condensates under low ionic strength conditions under the addition of SNARF-4F to read out pH. Image obtained by emission detection in 580 nm (left) and 650 nm (middle) as well as feature segmentation of the individual condensates including annotation (right). (**e**) SNARF-4 F 580 nm/650 nm emission ratio plotted against the number of pixels for each individually analyzed PGL3 condensate. Light-shaded background points represent individual data, while magenta symbols and error bars show mean ± SD within each bin of pixel ranges delineated by dashed vertical lines; where for the ranges from left to right n = [20, 14, 11, 10, 7, 14, 10, 10, 7, 4]. (**f**) Condensate pH, derived from the average fluorescence ratio, plotted against the effective droplet diameter (assuming spherical geometry). Data are represented as mean ± SD with the sample size from left to right: n = [20, 14, 11, 10, 7, 14, 10, 10, 7, 4]. Source data

We next set out to orthogonally confirm the observation of pH gradients between the dense and dilute phase. We applied the ratiometric pH-sensing dye SNARF-4F and read out the dye signal in a spatially resolved manner using confocal imaging (Extended Data Fig. 5). We find that while the dilute-phase pH varies with changing the initial condition, the dense-phase pH remains constant at ~6.5–7 (Fig. 1f), corroborating the observations from the tie-line-based approach. Indeed, unmodified insulin samples show a similar trend where the dense phase pH is constant with varying initial conditions (Extended Data Fig. 4c,d). As such, the condensed phase effectively appears to act as a spatially localized buffer that can counteract the environmental pH condition. Such protein buffering is expected, given common dense-phase concentration in the low mM range^34–36^, meaning that the side-chain concentration will be comparable to or even exceed the environmental buffering. Further applying our tie-line-based approach, we found that dense-phase pH regulation is reinforced by the exclusion of small-molecule buffers, thereby favouring buffering by protein-associated ionizable groups (Extended Data Fig. 3 and Supplementary Notes).

### pH gradients mediate reduction of intermolecular electrostatic repulsion

We next examined how the dense-phase repulsion free energy changes with pH. As the repulsion free energy of a charged sphere of fixed radius scales with the square of its charge density^37^, we reason that the dense-phase repulsion free energy similarly scales with the square of the sequence charge density (*q*^2^). While factors such as dense-phase concentration also modulate this energy, *q*^2^ provides a useful proxy for relative energetic changes. The sequence charge density at varying pH was then calculated using tabled p*K*_a_ values of the individual amino acids, followed by normalizing by the sequence length *N* (*q* = *Q*/*N*). The *q*^2^ profile for insulin glargine shows a minimum around the pI, with a sharp increase at around pH 5 and 8 (Fig. 1g), corroborating that the observed pH gradients help mitigate electrostatic repulsion in the dense phase. The repulsion profile also resembles the overall phase boundary, which is similarly observed for human insulin.

To test this further, we sought to modulate the electrostatic repulsion through addition of potassium chloride (KCl) and associated charge screening. At fixed KCl concentrations of 75 mM and 150 mM, an increase in the width of the pH-responsive phase boundary for insulin glargine was observed (Fig. 1h). Hence, phase separation could occur at higher polypeptide net charge owing to the added charge shielding. Differential shifts of the boundary in the high-pH regime compared with the low-pH regime are likely associated with ion-side chain-specific interactions or potential charge regulation effects aided by the presence of ions.

To quantify counter-ion effects, we applied the previously described energy dominance analysis approach^18,31,32^, which maps the relative free energy gain (*D*_i_) of individual components to the overall phase separation driving force. Tracking insulin glargine concentration trajectories at fixed pH (Extended Data Fig. 2d), we evaluated the dilute-phase response gradient (*R*_AA_) to derive dominance (*D* = 1 – *R*). At pH 6.1 without salt, insulin glargine shows *D*_p_ = 0.46, indicating additional contributors aside from the peptide to the free energy gain. *D*_p_ peaks near the peptide pI (Fig. 1i), consistent with more one-component-like behaviour at minimal net charge. With added KCl, this shape is conserved, but *D*_p_ decreases as ions increasingly contribute to the energetic driving force.

### PGL3 condensation creates acidic pH microenvironments

**Extended Data Fig. 6:**
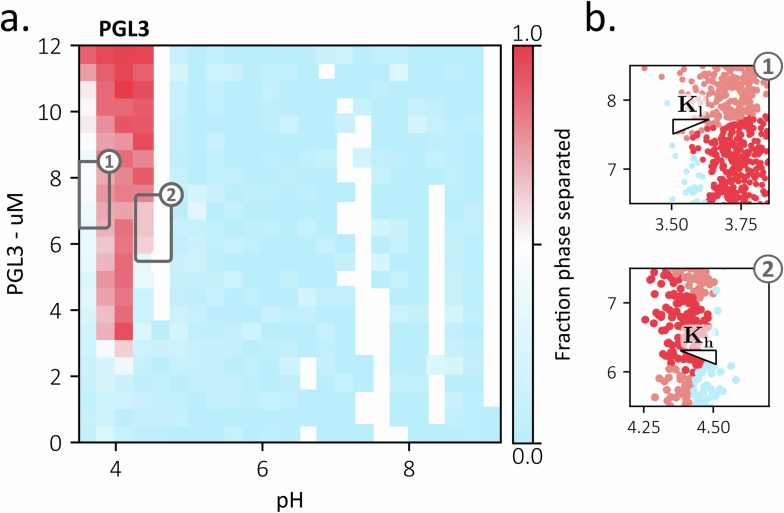
pH-responsive phase behavior for PGL3. (**a**) Phase diagram for PGL3 as obtained from the combinatorial droplet microfluidic approach. The red-to-blue gradient indicates the fraction of phase separated conditions obtained in a particular bin. (**b**) Zoom in of (**a**) displaying the individual data points and visualising the dilute phase contour gradient. Source data

**Extended Data Fig. 7:**
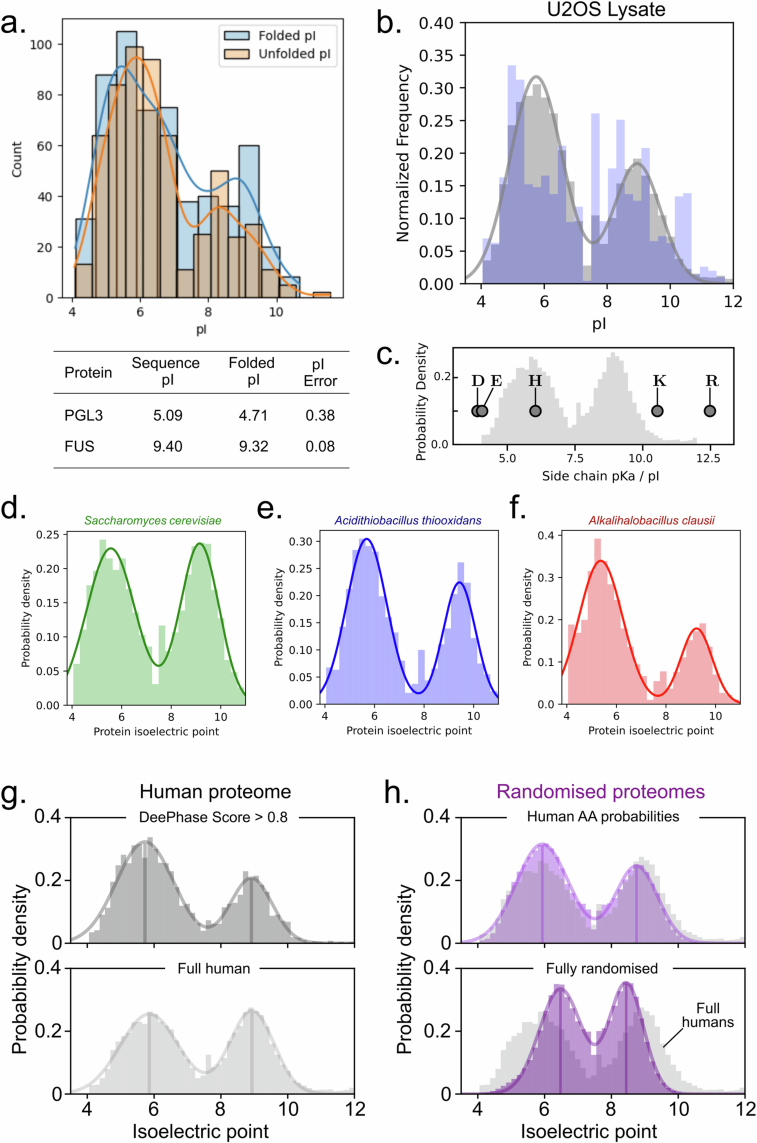
Electrostatic properties of proteomes. (**a**) The plot displays the pI histogram of a subset of SwissProt/UniProt Human proteins using consideration of the folded state via application of the PypKA software^5^ (blue) as well as from direct prediction of just the linearized sequence (orange). Table shows changes in the pI when considering folded features with PypKA^5^ for PGL3 and FUS. (**b**) pI density distribution of all proteins detected in U2OS cell lysate both when considering only the individual proteins (grey) as well as their relative abundance (blue), data from^6^. (**c**) Side chain pKa’s of Aspartic acid, Glutamic acid, Histidine, Lysine and Arginine compared to the pI distribution of the full human proteome (grey background). (**d**-**f**) pI distribution of the full set of individual proteins contained in the proteome of the yeast species Saccharomyces cerevisiae (**c**, green), as well as the extremophiles Acidithiobacillus thiooxidans (**d**, blue) and Alkalihalobacillus clausii (**e**, red) as obtained from Uniprot. (**g**) Distribution of isoelectric points of highly phase separation prone proteins (DeePhase Score > 0.8)^7^ (top) as well as the full human proteome (bot). (**h**) Distribution of isoelectric points of randomized proteomes compared to the human proteome. (top) Human amino acid abundance probability and length distribution and (bot) fully randomized sequences. Source data

We reasoned that condensate pH gradients driven by charge neutralization should extend to other protein systems. To test this, we investigated the condensation of PGL3, a protein critical to the germ cell development of *Caenorhabditis elegans* by scaffolding the formation of P-granules^38^. Similarly, to the insulin system, PGL3 phase separation occurred near its pI (Extended Data Fig. 6; pI_PGL3_ = 5.1 from sequence-based prediction with minimal changes owing to folding; Extended Data Fig. 7a). Tie-line analysis further indicated a shift of the dense-phase pH towards more acidic values close to the pI (Extended Data Fig. 6), highlighting that dense-phase pH gradients are also observed for more complex protein systems.

**Fig. 2:**
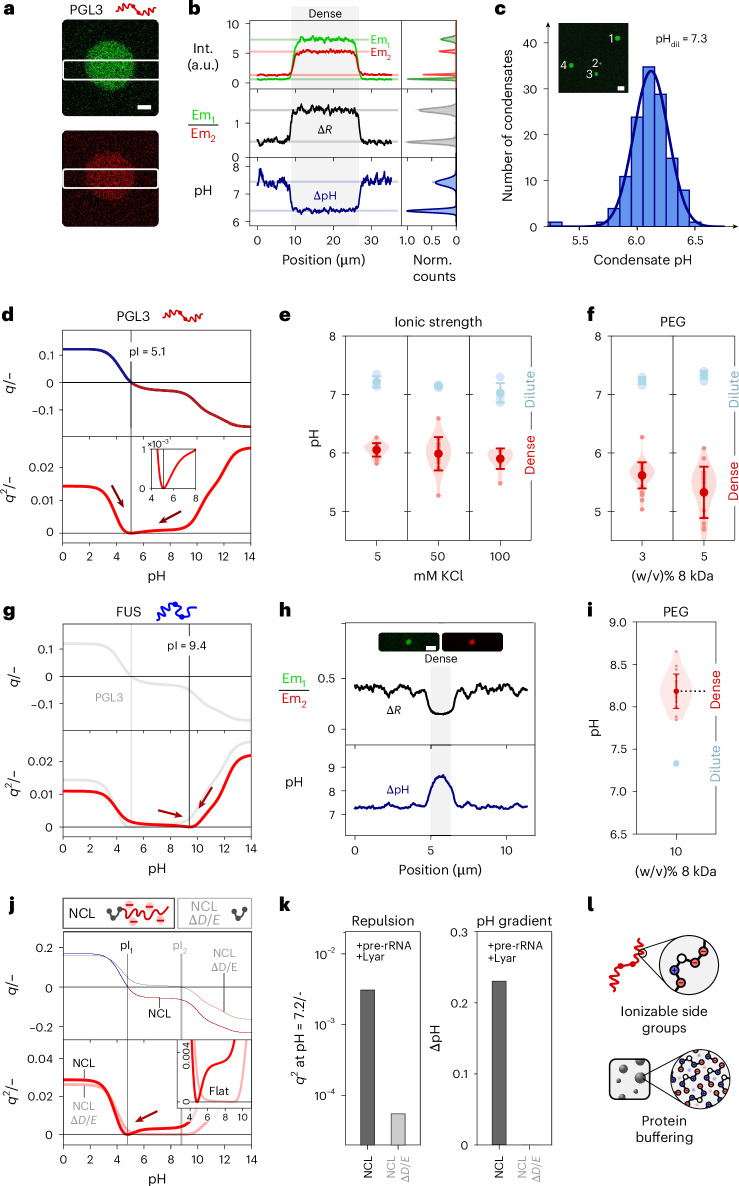
Biomolecular condensates sustain dense-phase pH gradients buffered towards the protein pI. **a**, Confocal imaging of PGL3 condensates at 2 μM protein concentration and 1 mM KCl, 50 mM TRIS buffer at pH = 7.3 using the ratiometric pH-sensing dye SNARF-4F. The white outlines mark the part of the image used to generate **b**. Scale bar, 5 μm. **b**, Spatially resolved ratiometric dye signal in the dense and dilute phase for both emission channels, the emission intensity ratio and the corresponding pH, including readout histograms of all signal traces. **c**, Condensate pH distribution for large numbers of PGL3 condensates formed at low ionic strength conditions from **a**. The dilute-phase pH was measured at 7.3. Inset: confocal image of multiple condensates. Scale bar, 20 μm. **d**, Net charge density and net charge density square of PGL3 protein with varying pH, with a minimum close to the pI as illustrated with the arrows. **e**,**f**, PGL3 dense- and dilute-phase pH at varying KCl (**e**) and PEG (**f**) concentrations. Fixed total protein concentration and buffer condition (2 μM protein, pH = 7.3, 50 mM TRIS buffer, 150 mM KCl in **g** and **h**). Dilute-phase pH data are presented as mean ± s.d. from *n* = 3 individual technical replicates. Dense-phase data points represent the pH of individual condensates overlayed with the obtained mean ± s.d. and shaded violins highlighting the distribution. **g**, Net charge density and net charge density square of FUS protein with varying pH, with a minimum close to the pI as illustrated with the arrows. **h**, Spatially resolved ratiometric dye signal in the dense and dilute phase for both emission channels, the emission intensity ratio and the corresponding pH, for a FUS condensate formed at 10% (w/v) PEG, 2.5 μM protein, 150 mM KCl and 50 mM TRIS buffered at pH = 7.3. Scale bar, 2 μm. **i**, FUS dense- and dilute-phase pH at 10% (w/v) PEG, 2.5 μM protein, 150 mM KCl and 50 mM TRIS buffered at pH = 7.3. The data are represented in the same way as detailed in **e**. **j**, Comparison of net charge density and net charge density square for the NCL protein and Δ*D*/*E* NCl deletion mutant (deletion of a sequence region rich in acidic amino acids – *D*/*E* tract). The arrow indicates the difference in minima based on the pI. **k**, Comparison of the net charge density square at pH = 7.2 for NCL with and without the *D*/*E* tract (left). Observed in vitro pH gradient from ref. ^25^ for condensates formed with 15 μM NCL protein, 1 μM pre-rRNA and 0.2 μM Lyar protein. **l**, Schematic representation of the mechanism of action underlying the protein dense-phase buffering that drives pH gradients. Int., intensity; Norm., normalized. Source data

We then sought to assess the impact of the phase separation trigger: when induced PGL3 phase separation by decreasing the ionic strength at fixed pH (50 mM TRIS, pH = 7.3), we also observed an acidification of the dense phase compared with the dilute phase (Fig. 2a–c). This effect was also independent of condensate size (Extended Data Fig. 5e,f). This pH shift decreases intermolecular PGL3 repulsion, as confirmed by a minimum in the *q*^2^ profile at acidic pH (Fig. 2d). The apparent dense-phase pH of ~6–6.5, slightly above the pI, likely stems from the asymmetric *q*^2^ profile and partial electrostatic screening by ions.

To probe the influence of solutes, we varied KCl and polyethylene glycol (PEG) concentrations during PGL3 phase separation (Fig. 2e,f). Across 5–100 mM KCl, the dense-phase pH remained below 6.5, indicating that the pH gradient persists regardless of ionic strength. Likewise, PEG-induced condensation yielded an acidic dense phase near the pI, demonstrating that maintaining a dense-phase pH gradient is a general requirement for homotypic PGL3 phase separation, independent of salt concentration or triggering mechanism.

### Charge density governs condensate pH gradients

**Extended Data Fig. 8:**
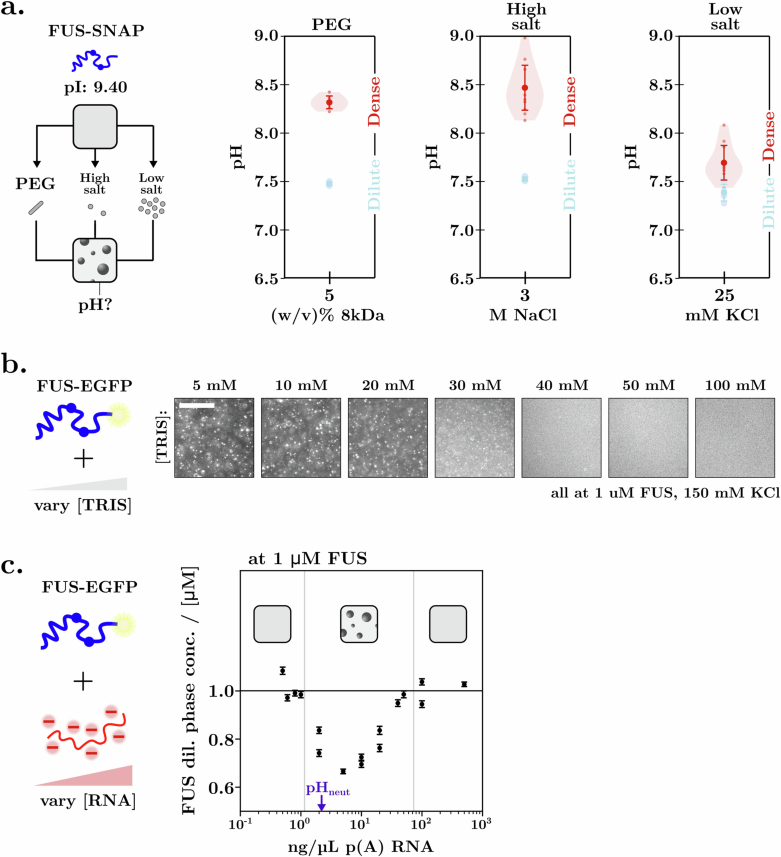
Effect of environmental conditions and RNA on FUS phase separation behavior. (**a**) Dense and dilute phase pH readout of condensates formed from FUS-SNAP using PEG, high salt and low salt conditions as phase separation triggers. Experiments were performed at fixed total protein concentration (2 μM) and buffer conditions (pH=7.3, 50 mM HEPES buffer) and 150 mM KCl in the PEG sample. Dilute phase pH data is presented as mean ± SD from n > 3 individual repeats. Dense phase data points represent the pH of individual condensates overlayed with the obtained mean ± SD and shaded violins highlighting the distribution. (**b**) Effect of varying the buffer strength on FUS phase behavior, highlighting that phase separation is observed at low TRIS concentrations. Widefield imaging of FUS-EGFP condensates under varying concentrations of TRIS buffer at constant pH = 7.3 as well as constant total protein concentration (1 µM) and salt conditions (150 mM). (**c**) Changes in the dilute phase concentration of FUS condensation with varying p(A)-RNA concentrations. Phase separation is observed near composition at which the net charge of the pK + PGL3 mixture is predicted to be neutral at physiological pH (purple arrow). Data are represented as the mean ± SD from n > 3 repeats. Source data

Based on our observations highly positively charged proteins are expected to similarly form alkaline condensate pHs. To test this, we turned to FUS (pI = 9.4; Fig. 2g), a protein implicated with aberrant phase transitions in the emergence of amyotrophic lateral sclerosis^39^. Upon PEG-induced phase separation, SNARF-4F readouts revealed an apparent dense-phase pH >8 (Fig. 2h,i). Similar to PGL3, FUS sets up a pH gradient towards its pI under a range of other homotypic phase separation conditions (Extended Data Fig. 8a), highlighting that condensates can sustain pH buffering towards a variety of pH conditions. The condensed-phase buffering effect is corroborated by an observed competition between FUS phase separation and the environmental buffer (Extended Data Fig. 8b). Notably, condensation is induced by decreasing buffer concentrations, as the condensed phase can more easily regulate its pH.

To investigate how the effective protein–protein repulsion modulates condensate pH gradients, we turned to previously published work on an NCL protein system associated with nucleolar phase separation^25^ (Fig. 2j). The negatively charged wild-type NCL protein (pI = 4.8) forms a slightly acidic dense phase when undergoing phase separation with RNA, in the presence of low concentrations of another client protein (LYAR, 70-fold lower concentration). This is consistent with NCL’s *q*^2^ profile with a clear minimum at low pH (Fig. 2k). Deletion of a region rich in negatively charged amino acids from the protein (Δ*D*/*E* NCl, pI = 8.8) removes the presence of a dense pH gradient. Despite its higher pI, this variant exhibits near-zero net charge between pH 5 and 9 (Fig. 2j), resulting in minimal repulsion even away from the pI. These findings indicate that pH gradients arise only when sufficient electrostatic repulsion is present under environmental conditions (Fig. 2l).

### Effect of RNA scaffolding on condensate pH gradients

**Fig. 3:**
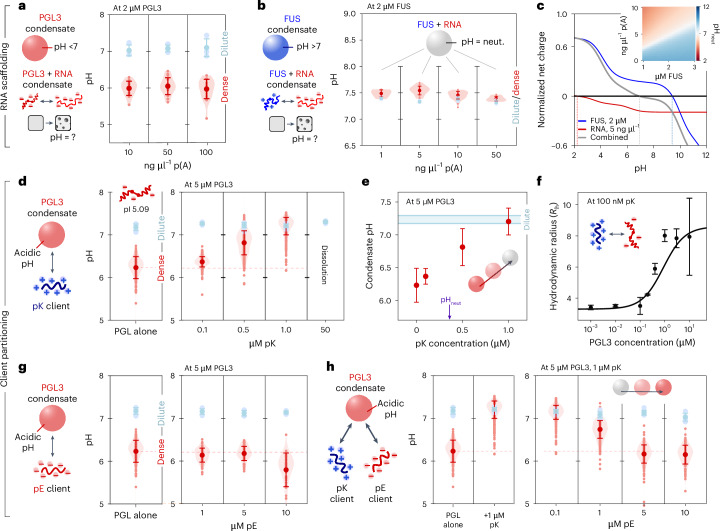
Condensate pH can be dynamically engineered through composition. **a**, PGL3 dense- and dilute-phase pH at varying KCl and PEG concentrations. Fixed total protein concentration and buffer condition (2 μM protein, pH = 7.3, 50 mM TRIS buffer, 150 mM KCl in **g** and **h**). Dilute-phase pH data are presented as mean ± s.d. from *n* > 3 technical replicates. Dense-phase data points represent the pH of individual condensates overlayed with the obtained mean ± s.d. and shaded violins highlighting the distribution. **b**, FUS–RNA co-condensate pH at varying p(A)-RNA concentrations. While homotypic FUS condensates show an alkaline dense-phase pH, heterotypic FUS–RNA condensates show neutral dense-phase pH across a range of conditions. The data are represented in the same way as detailed in **a**. **c**, Net charge change of FUS with varying pH compared with p(A)-RNA and a FUS/RNA mixture. The normalization is performed to account for the concentration difference between RNA and protein, followed by bounding to the maximum FUS charge. Dashed lines reflect the pI of the three observed pH-dependent charge curves. Inset: heat map showing the pH at which neutral net charge is reached for different FUS/RNA compositions. **d**, Addition of poly-lysine (pK) to formation of PGL3 condensates and observed dense-phase pH values. The data are represented in the same way as detailed in **a**. **e**, Change in dense-phase pH with increasing pK concentration. Blue range: indicates the dilute-phase pH. Purple arrow: composition at which the net charge of the pK + PGL3 mixture is predicted to be neutral at physiological pH. Data are represented as the mean ± s.d. from **d**. **f**, Quantification of monomer binding between FITC-tagged pK and PGL3. Hydrodynamic radiusmeasurements prvoided in nm are performed using microfluidic diffusional sizing. Data are represented as the mean ± s.d. from *n* = 3 technical replicates. **g**, Effect of poly-glutamic acid (pE) to formation of PGL3 condensates and observed dense-phase pH values. The data are represented in the same way as detailed in **a**. **h**, Simultaneous addition of pE and pK to PGL3 condensates. Addition of positively charged pK neutralizes the acidic PGL3 condensate pH. Upon increasing addition of pE at constant PGL3 and pK concentrations, the condensate pH is shifted back to acidic conditions. The data are represented in the same way as detailed in **a**. In **d**, **g** and **h**, the red dashed line shows the comparison to the average condensate pH for the single component PGL3 condensate. ?, unknown; neut., neutral. Source data

Protein phase separation commonly arises from heterotypic interactions, especially with nucleic acids, leading us to further investigate the effect of heteromolecular mixtures on the condensate pH. When triggering PGL3 phase separation by addition of polyadenylic acid RNA (p(A)-RNA), we find an acidic apparent pH_den_ of around 6 (Fig. 3a) comparable to homotypic PGL3 condensates (Fig. 2c,e). Crucially, nucleic acids are also characterized by a low pI because of their phosphate backbone groups. Therefore, heterotypic PGL3–RNA interactions will also become more favourable at lower pH because of a decrease in repulsion. The co-partitioned RNA will also contribute to decreasing the dense-phase pH via buffering of its phosphate backbones.

We next set out to study the effect of scaffolding between the positively charged FUS protein and negatively charged RNA. For FUS phase separation driven by interaction with p(A)-RNA, no alkaline pH gradient is observed contrary to homotypic conditions (Fig. 3b). Here the formation of a pH gradient is no longer required because the heterotypic FUS–RNA scaffold enables attraction even at environmental pH. The resulting condensate adopts an effective pI tied to the stoichiometry of the FUS and RNA interaction. Indeed, mixtures of RNA and FUS reach charge neutrality close to pH = 7 under concentration conditions comparable to our experiments (Fig. 3c). When the RNA concentration is varied, the pH of FUS–RNA condensates remains unchanged, implying stabilization around a fixed, charge-neutral stoichiometry. Measurements of the dilute-phase FUS concentration confirm this, as phase separation is maximized at stoichiometries close to predicted charge neutrality (Extended Data Fig. 8c).

### Tuning condensate pH gradients via client partitioning

Proteins can also passively partition into condensates without triggering phase separation or providing scaffolding. Clients with distinct electrostatic properties, therefore, might enable modulation of the electrostatic repulsion and thus the dense-phase pH. To test this, we next subjected PGL3 condensates formed through low ionic strength conditions to addition of highly charged poly-amino acid clients (poly-lysine, pK, and poly-glutamate, pE). Upon addition of the positively charged pK, the acidic pH gradient observed for PGL3 condensates decreased (Fig. 3d), even scaling linearly with pK concentration until effective abrogation of the pH gradient (Fig. 3d,e). Notably, a higher concentration of pK than predicted for charge neutrality was required to achieve neutral condensate pH, likely owing to incomplete pK partitioning (Fig. 3e, purple arrow). Beyond this point, phase separation ceased because saturative pK and PGL3 binding prevented PGL3–PGL3 network formation required for condensation (Fig. 3f).

We then proceeded to add negatively charged pE to acidic PGL3 condensates. Here while limited effects were observed, at high pE concentrations, the condensate pH decreased even below the conditions formed by PGL3 alone. To study the effects of multiple clients, we then also added both pE and pK to PGL3 condensates starting from PGL3 and pK conditions where condensates showed no pH gradient. Crucially, upon addition of pE, the dense-phase pH continuously decreased towards the acidic conditions observed for homotypic PGL3 condensates. Thus, our data highlight that client addition can enable precise control over the dense-phase pH through altering the effective electrostatic component mixture.

### Multiphasic, multi-pH condensates

**Fig. 4:**
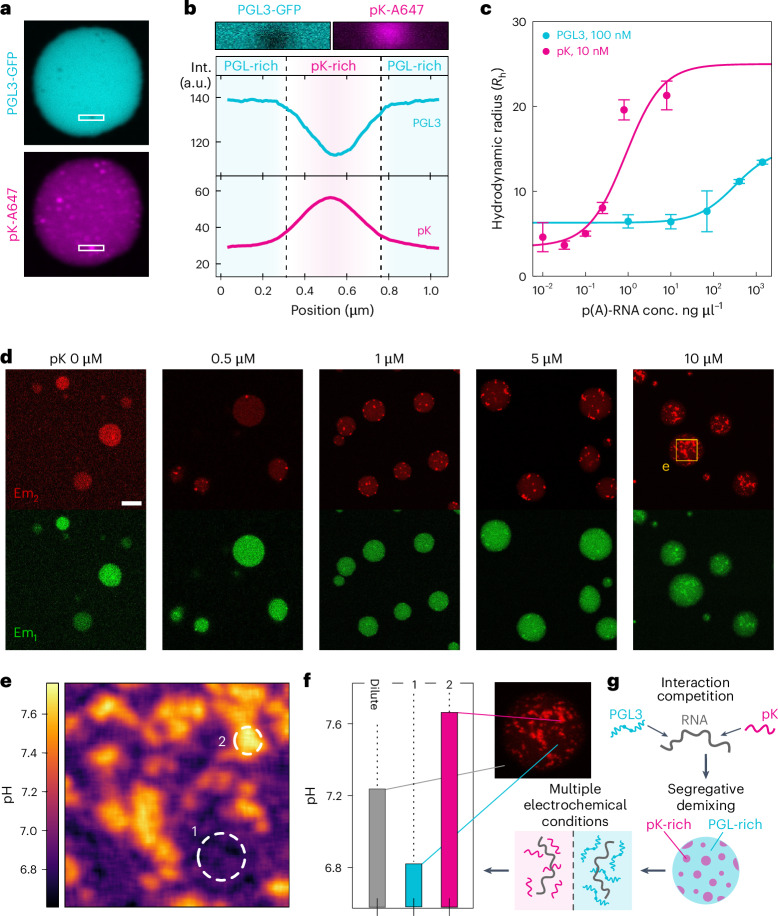
Multiphase, multi-pH condensation of PGL3 with p(A)-RNA and poly-lysine (pK). **a**, Confocal images of PGL3, pK and p(A)-RNA condensates, with covalent fluorescent labels on PGL3 (cyan) and p(K) (magenta). **b**, Fluorescence intensity profiles across a multiphasic condensate sub-feature, indicating distinct pK- and PGL3-rich domains. **c**, RNA binding curves for PGL3 and FITC-tagged pK, obtained by monitoring hydrodynamic radius (provided in nm) changes via microfluidic diffusional sizing. Data are represented as the mean ± s.d. from *n* = 3 technical replicates. **d**, SNARF-4F-based pH imaging of PGL3–pK–RNA condensates at different pK concentrations. Scale bar, 5 µm. **e**, Spatially resolved pH map of a multiphasic condensate from **d**, highlighting alkaline (pK-rich) and acidic (PGL3-rich) regions. **f**, Quantitative comparison of local pH values in distinct multiphasic sub-features relative to the dilute phase. **g**, Schematic illustrating competitive RNA binding to RNA between PGL3 and pK, which drives the formation of segregated dense phases with unique electrochemical microenvironments at equilibrium. Source data

We next examined client partitioning in protein–RNA condensates by forming PGL3–RNA condensates, which are acidic in the absence of clients, while adding positively charged pK. This produced multiphase condensates with distinct PGL3- and pK-rich phases (Fig. 4a,b). Binding affinity measurements showed that pK binds p(A)-RNA with much higher affinity than PGL3 (Fig. 4c), explaining the multiphase behaviour: both proteins compete for RNA binding sites, favouring separate pK-rich domains where pK–RNA interactions dominate. Indeed, increasing the pK concentration led to increased numbers of multiphasic structures (Fig. 4d). Using SNARF-4-based pH readouts (Fig. 4d,e), we observed that each phase showed its own pH. The pK-rich phase was slightly alkaline, whereas the PGL3-rich phase was slightly acidic, reflecting their opposing electrostatic properties (Fig. 4f). Thus, condensates not only maintain a pH gradient relative to the dilute phase but can also form multiphasic, multi-pH architectures via segregative demixing (Fig. 4g).

### Phase-separating proteins in general are highly charged

**Fig. 5:**
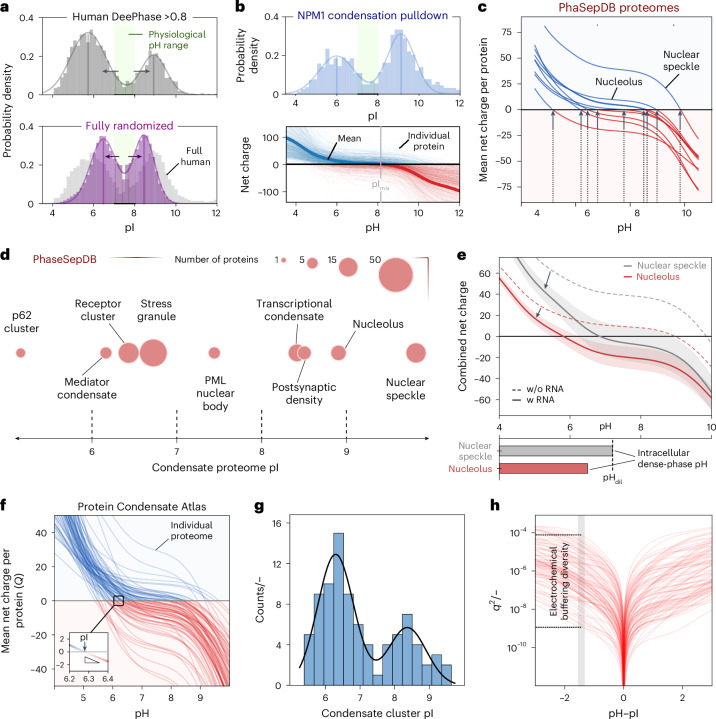
Generic protein charge bias translates to intracellular condensate pH gradients and massive electrochemical diversity of human condensates. **a**, pI distribution of highly phase separation-prone proteins as quantified using DeePhase Scoring (DeePhase Score >0.8)^42^ (top) compared with fully randomized sequences and the human proteome (bottom). **b**, pI distribution of quantitative proteomics data obtained from NPM1 condensation in cellular lysate from ref. ^43^ (top). Net charge change with pH of the full set of individual proteins obtained from **b** compared with the mean. The point at which the mean net charge curve crosses the *x* axis is referred to as the condensate mixture pI (bottom). Regarding correction for the relative abundance of proteins, see Extended Data Fig. 9. **c**, Visualization of condensate proteome net charge change with pH for complex condensate proteomes obtained from PhaSepDB^44^. **d**, Comparison of condensate proteome mixture pIs of complex condensate proteomes as obtained via evaluation of protein sequences from PhaSepDB^44^. **e**, Comparison of nucleolus and nuclear speckle net charge density square profiles and correlation to observed in vivo pH gradient from ref. ^25^. Dashed lines show the average net protein charge calculated without RNA. Solid lines incorporate a 7.5% RNA mass fraction, with the arrows indicating the associated shifft and shaded regions showcasing the 5–10% RNA mass fraction range, consistent with published nucleolar fractionation data^45–47^. **f**, Schematic representation of the Protein Condensate Atlas from ref. ^48^ used to evaluate the theoretical range of electrochemical properties presented by complex condensates. **g**, Histogram of condensate cluster pIs obtained for all 85 entries from the Protein Condensate Atlas. **h**, Comparison of the net charge density square for the Protein Condensate Atlas. Grey box: highlighting the range of *q*^2^ values of different condensate clusters at similar pH–pI. PML, promyelocytic leukaemia. Source data

Our data suggest that proteins with distinct physico-chemical properties can set up diverse pH gradients. To assess the broader relevance, we analysed the pI distribution of the human proteome (*n* = 20,324), revealing a pronounced bimodal shape largely devoid of proteins with near-neutral charge (7 < pI < 8; 8.5%; Fig. 5a). While this charge bias is likely associated with guaranteeing cytosolic solubility, it highlights that proteins are in general highly charged. Here folding effects (Extended Data Fig. 7, probed by application of the PypKA software^40^) and cellular expression levels (Extended Data Fig. 7a, as shown by mass spectrometry of U2OS cell lysates^41^) were shown to only have a small effect on the overall distribution. The persistence of this charge diversity implies a potentially widespread requirement for adjusting dense-phase repulsion through pH gradients.

We then sought to understand this charge bias specifically for phase separation-prone proteins by applying a filter of high phase separation propensity using DeePhase^42^ (score between 0 and 1; threshold >0.8, *n* = 3,950). We found a nearly identical pI distribution (7.2% with 7 < pI < 8), suggesting that electrostatic diversity and the potential for condensate pH regulation are general features of biomolecular condensation. A disparity in the probability density between acidic and basic pI values can potentially be attributed to a bias of DeePhase for homotypic phase-separating proteins as high-pI proteins are more likely to engage in protein–RNA interactions.

The bimodal pI distribution likely originates from the charge properties of natural amino acids, as none achieve neutrality at physiological pH (Extended Data Fig. 7b), nor do most combinations of them. Consequently, proteomes across taxa—including extremophiles adapted to harsh environments—exhibit similar charge biases (Extended Data Fig. 7c–e). The bimodal pattern also persists in randomized sequences (Fig. 4a and Extended Data Fig. 7f,g), with human amino acid abundances further skewing towards higher net charge. Thus, pH gradients during condensation processes likely represent a generic feature rooted in the fundamental properties of amino acids.

### In silico evaluation of the electrochemical properties of complex condensates

**Extended Data Fig. 9:**
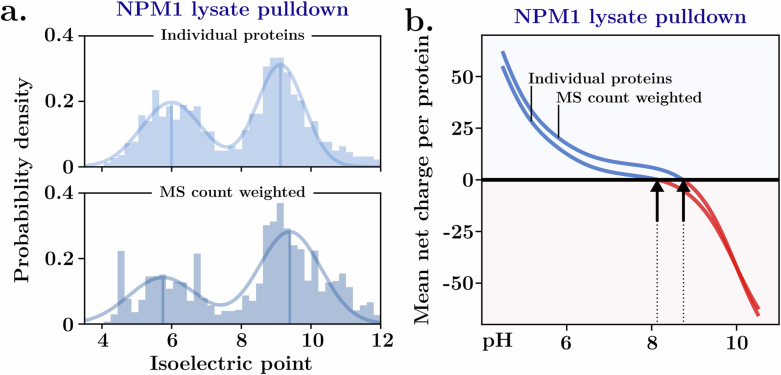
Electrochemical properties of an NMP1 lysate pulldown proteome. (a) Distribution of isoelectric points of quantitative proteomics data obtained from NPM1 condensation in cellular lysate^6^ both considering only the individual proteins (top) and the relative abundance (bot). (**b**) Changes in the net charge of the NPM1 lysate pulldown proteome with varying pH as normalized by the total number of proteins, showing the comparison between considering just the individual proteins as well as the quantitative abundance data. The point at which the condensate proteome net charge is neutralized is further referred to as the condensate proteome mixture pI. Source data

In more complex environments, condensates commonly recruit numerous client proteins, all of which could contribute to the electrochemical properties of the condensate. As an example, we analysed published proteomics data of an NPM1 condensation pulldown in cellular lysate, mimicking the composition of granular compartments inside the nucleus^43^. To infer information on the electrochemical properties, we considered the pI distribution of the individual proteins in the condensate proteome (that is, the collection of sequences recruited into the condensate). While NPM1 itself is acidic, the condensate proteome is enriched in high-pI proteins (Fig. 5b and Extended Data Fig. 9), and this positive bias increases further when weighted by abundance (proteins with pI > 7, 63% → 68%). Hence, condensate electrochemical properties strongly depend on client recruitment, consistent with our experimental observations. Such charge bias likely reflects selective partitioning of positively charged proteins into nucleic acid-rich environments, promoting further recruitment of similarly charged clients.

To characterize the electrochemical behaviour of this condensate, we averaged the net charge–pH profiles of all recruited proteins within the condensate proteome (and identified an effective mixture pI of ~8.5; Fig. 5b, bottom panel). Close correlation between unweighted (pI = 8.2) and abundance correlated data (pI = 8.7) suggests that the approximation of assessing only the properties of the unique sequences recruited functions well as a proxy in this case (Extended Data Fig. 9). Applying this analysis to PhaSepDB datasets^44^ yielded consistent proteome pIs for nuclear condensates, with the nucleolus (pI = 8.9) matching the NPM1 pulldown mimic (Fig. 5c,d), supporting the robustness of this electrochemical profiling approach.

To relate these in silico predictions to intracellular measurements, we compared them with reported pHluorin2 readout data for nucleoli and nuclear speckles^25^. Both condensate systems exhibit overall positively charged proteome pIs (Fig. 5d,e). Yet recruitment of negatively charged nucleic acids is expected, which we accounted for by assuming an approximate mass fraction of 5–10% in complex condensates^45–47^. Accounting for both protein and nucleic acid contributions, nuclear speckles are predicted to reach neutrality near pH 7, which coincides with no pH gradient detected in cellulo (Fig. 5e). Nucleoli, however, become neutral only below pH 7, consistent with their observed slightly acidic microenvironment (Fig. 5e). Thus, in silico electrochemical profiling of condensate proteomes accurately reflects their measured pH characteristics in cells.

### Dynamic electrochemical microenvironments through complex condensation

We next characterized the potential effector range and functional diversity of condensate electrochemical gradients. Even the mapped condensate proteomes from PhaSepDB show substantial variation in electrochemical properties (Fig. 5c,d): for example, stress granules exhibit a slightly acidic mixture pI of 6.8, whereas nuclear compartments are more basic. Across the top nine membraneless organelle systems, condensate proteome pI values span nearly five pH units (Fig. 5d), highlighting extensive electrochemical diversity in vivo.

**Extended Data Fig. 10:**
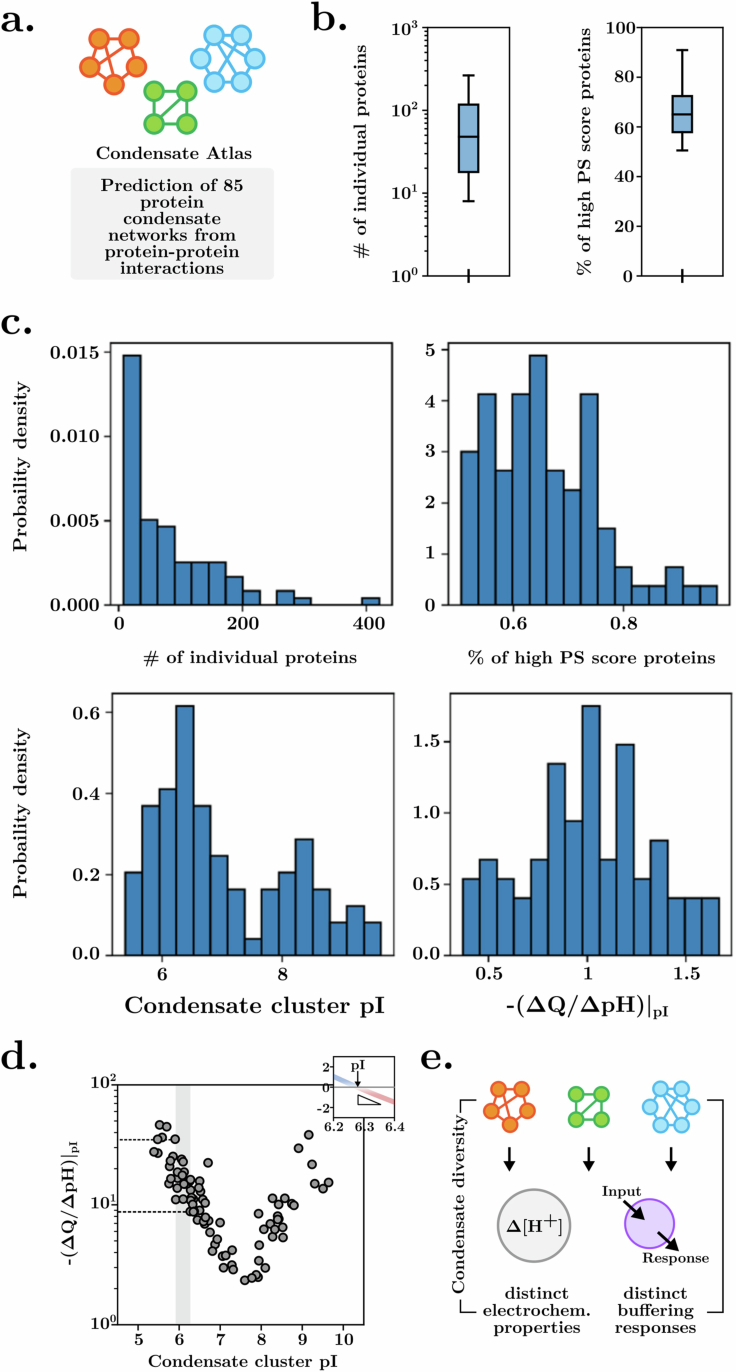
Property comparison of the Condensate Atlas. (a) Schematic representation of the Condensate Atlas and (**b**) box plot representation of the number of individual proteins per individual condensate network and percentage of phase separation prone proteins per individual condensate network in the Condensate Atlas (n = 85). Box plots are represented as median and 25^th^ and 75^th^ percentiles while the whiskers display the minimum and maximum values within 1.5× the interquartile range, outliers beyond this range are not displayed. (**c**) Histogram distributions of characteristic features of the Condensate Atlas. Histograms of the number of individual proteins per condensate network, fraction of phase separation prone proteins, condensate cluster pI and the net charge change at the pI (from left to right) for all 85 entries. All histograms given in probability density. (**d**) Evaluation of the proteome pH response behavior around the mixture pI. Change in the charge responsiveness to pH pertubations –(ΔC/ΔpH)|_pI_ against condensate cluster pI. Grey box: highlighting the range of –(ΔC/ΔpH)|_pI_ values of different condensate cluster with similar pI. (**e**) Schematic representation highlighting the diversity of potential pH gradients and pH buffering responses display by complex condensates. Source data

To explore this further, we analysed the Protein Condensate Atlas, which comprises 85 condensate clusters enriched in phase-separating proteins derived from unsupervised clustering of a protein–protein interaction network^48^ (Extended Data Fig. 10). These proteomes show mixture pI values spanning pH 5–10 (Fig. 5f,g), suggesting that condensates could potentially allow for a large diversity of dynamic pH microenvironments. The mixture pI distribution closely resembles the bimodal shape of the human proteome, likely reflecting protein abundance bias and a preference for condensates less dependent on protein–RNA interactions.

We then also sought to evaluate the response behaviours to environmental perturbations by assessing the change in the *q*^2^ repulsion proxy with variations in the pH. We found that the *q*^2^ term can vary up to 5 orders of magnitude across the 85 condensate clusters when only moving ~1.5 units away from the respective mixture pI. Hence, these systems show distinct electrostatic response behaviours to environmental perturbations. The slope of net charge change at the mixture pI, –(Δ*C*/ΔpH)_pI_, varied by a factor of 5 among clusters with pI ≈ 6 (Extended Data Fig. 10) alone. This suggests that some condensate systems can maintain their charged state across pH fluctuations, whereas others undergo switch-like transitions.

### Condensates as spatially compartmentalized buffers

**Fig. 6:**
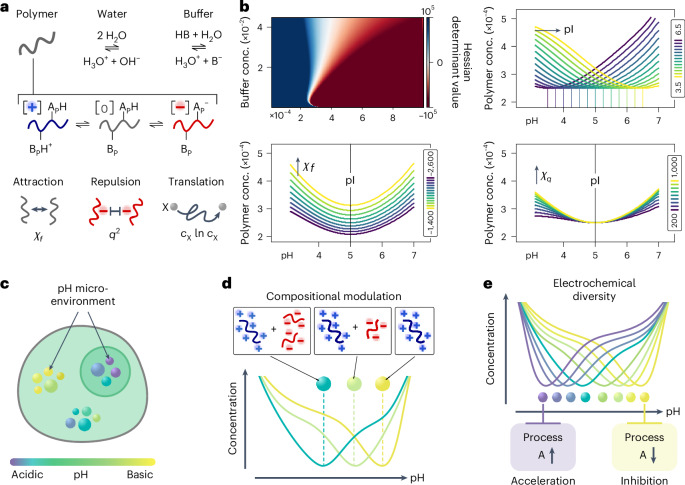
Biomolecular phase separation as a mechanism to generate distinct pH microenvironments. **a**, Theoretical model set-up to calculate the pH-dependent phase behaviour of a generic polymer. **b**, Prediction of composition-dependent spinodal positions from solving for the theoretical model and variation of key parameters in the pI, attractive Flory and repulsive electrostatic parameter. All concentrations are given in M. Top, left: Hessian determinant values (red-to-blue colour code) with varying system compositions at a fixed pI, attractive Flory and repulsive electrostatic parameter. Top, right and bottom row: individual curves represent spinodal positions as obtained by Hessian determinant evaluation. The colour code indicates variation of specified control parameters. **c**, To minimize electrostatic repulsion biomolecular condensates, sustain distinct pH microenvironments at equilibrium without necessitating membrane enclosure or the presence of active processes. **d**, Condensates pH and pH responsiveness can be dynamically fine-tuned through biomolecular properties, compositional changes or complex condensate architectures affecting the overall charge distribution. **e**, The diversity of electrochemical properties of complex condensates suggests potential for segregation and selective acceleration or inhibition of a range of functional processes, as highlighted visually by the modulation of a generic process A. Source data

Our data suggest that condensates maintain pH gradients to minimize electrostatic repulsion and that condensate-based electrochemical buffer systems may be widespread in nature. To further elucidate the origins of condensation-driven electrochemical buffering, we developed a minimal theoretical model in which a polymer can adopt positive, negative or neutral charge states within an aqueous, buffered environment (Fig. 6a). This model incorporates a generic attractive interaction that promotes phase separation, alongside electrostatic repulsion among charged states (see Appendix ‘Theoretical model’ for details). By analysing the Hessian, we identify regions of phase instability, that is, the spinodal. As expected, we found a minimum in the polymer’s critical concentration near a predefined pI, confirming that phase separation is most favourable when the net charge is minimized.

Increasing the generic attraction (decreasing the attractive parameter *χ*_f_) shifts the spinodal downwards, allowing phase separation at pH values farther from the polymer’s pI for a fixed polymer concentration (Fig. 6b). Conversely, stronger electrostatic repulsion narrows this minimum, requiring higher polymer concentrations to enable separation at pH values offset from the pI (Fig. 6b). In other words, the homogeneous phase becomes unstable primarily near conditions that reduce net electrostatic repulsion, driving the system towards a stable, high-polymer-density regime. In the resulting condensed phase, the pH will then be determined by the underlying acid–base equilibrium to which the high concentration of charged polymer side chains will contribute, while buffer species and other components can also partition differentially. Consequently, condensates effectively function as spatially compartmentalized buffers, forming and sustaining pH gradients at equilibrium.

## Discussion

Effects of pH on the rate of biological and chemical processes, by, for example, regulating enzyme activity or direct catalytic function of H^+^ and OH^−^, are well documented. Membrane-bound organelles, for example, commonly show distinct pH conditions aimed at optimizing processes relevant to their function, but require energy input to maintain^7–12^. In this study, we reveal that condensation presents a generic mechanism for the formation of pH gradients at equilibrium without necessitating active processes through spatially compartmentalized buffering.

Emergent properties arising from collective interactions have been a point of contention since the initial discovery of protein condensation. Our work details that condensates sustain pH gradients to mitigate electrostatic repulsion between charged proteins in the dense phase. This pH regulation is only possible through the formation of a distinct phase, which creates differential solute partitioning and high local protein concentrations to set up a distinctly buffered environment. In protein folding or aggregation, for example, charge neutralization is similarly required for assembly. However, the electrostatic repulsion commonly has to be overcome by shifts in the p*K*_a_ values of residues in the protein core or the assembly interface^49–54^.

While biomolecular condensation yields drastic increases in viscosity, the molecular-scale dynamics remain rapid enough to support efficient reactions^55^. As such, condensates have been shown to affect reaction rates^56–59^, and recent evidence highlights that such condensate pH gradients can modulate enzyme-catalytic outcomes^60,61^ or modulate redox reactions^24^. Such differential ion partitioning within condensates can also give rise to transmembrane-like interfacial potentials^62^. The observed large diversity of electrochemical properties presented by intracellular condensate networks (Fig. 6b), therefore, highlights the potential of biomolecular condensation to segregate and optimize functional processes through changes in the emergent electrochemical environment (Fig. 6c). This promise is further expanded by observations that coupling to non-equilibrium phenomena can even drive complex emergent behaviours such as induced microscopic flows^63,64^ pointing to a potential broader functional repertoire. Similarly, exploring how ageing or gelation processes modulate these pH gradients and associated functions will present an exciting area for future research.

Our data also highlight that control of the pH gradient arises from the physico-chemical properties of constituting biomacromolecules. This sets up potential for dynamic fine-tuning of the condensate pH through composition and client recruitment. Similarly, modulation of the protein properties through post-translational modifications^65–67^ such as phosphorylation is expected to play a role. Together, these dynamic modulation levers can enable precise control of the condensate pH. Combining this with the potential for multiple coexisting dense phases with distinct internal pH values could provide additional layers of regulation for sequential reactions or biochemical process cascades more generally.

This also has potential consequences to hypotheses that membraneless compartmentalization might have been an important factor in the origin of life^22,68^. Here, shifts in the pH of protocells formed through condensation of protobiomolecules could be a contributor to generating biological functionality and sustaining required pH conditions. Similarly, pH gradients arising from macromolecular density transitions could be leveraged in technological applications, for example, by building on the rich history of polymer or coacervate phase transitions, to generate designer condensation systems with optimal electrochemical properties for desired processes.

## Methods

### Reagents

All reagents and chemicals were purchased with the highest purity available. p(A)-RNA with a molecular weight range from 700 kDa to 3,500 kDa was purchased from Sigma-Aldrich as lyophilized powder and dissolved into a stock of 10 mg ml^−1^ in water before use. A 50% (w/v) PEG 8,000 Da stock solution was prepared by dissolving PEG pellets (Sigma-Aldrich) in 25 mM TRIS-HCl pH 7.3 buffer solution. The stock solution was stored at room temperature. Histidine and succinic acid were purchased from Sigma-Aldrich. Cy5-NHS ester was purchased from Thermo Fisher and dissolved in DMSO to give a 10 mM stock concentration and stored at −20 °C. SNARF-4F was obtained from Thermo Fisher and subsequently dissolved in DMSO to give a 10 mM stock concentration, which was stored at −20 °C. Insulin was purchased from Sigma-Aldrich and dissolved in high-pH H/S buffer (50 mM, pH = 9.0) to prevent precipitation at, for example, 5 mg ml^−1^. Insulin glargine was ordered from Sigma-Aldrich and dissolved in water (pH = 4, HCl adjusted) with 2% (w/v) glycerol, 0.2 mM ZnCl and 25 mM m-cresol. Poly-lysine (pK, 30–70 kDa), poly-lysine-FITC (30–70 kDa) and poly-glutamic acid (pE, 15–50 kDa) were ordered from Sigma-Aldrich and subsequently dissolved in the respective buffer used for the specified experiment.

### PGL3 protein expression

PGL3-6xHis-mEGFP purification was performed according to a published protocol^38^. Untagged PGL3 was then obtained by cleaving off 6xHis and mEGFP tags from the C terminus of PGL3-mEGFP-6xHis protein via usage of 6xHis-tagged TEV protease. Subsequent incubation with Ni-NTA agarose resin allowed for removal of 6xHis-tagged mEGFP and TEV protease. Further purification of untagged PGL3 was performed via size-exclusion chromatography using a HiLoad 16/60 Superdex 200 column (GE Healthcare) in 50 mM HEPES pH 7.4, 0.3 M KCl and 1 mM DTT. The protein was stored at −80 °C, followed by aliquoting and flash freezing in liquid nitrogen.

### FUS protein expression

FUS wild-type, EGFP and SNAP tagged variants were expressed as reported previously in an insect cell expression system^39,69^. After purification, the proteins were stored in 50 mM TRIS buffer (pH 7.3), 750 mM KCl, 1 mM DTT, 5% glycerol and at a total protein concentration of 20 µM and 70 µM for wild-type and SNAP variants, respectively.

### Histidine and succinic acid buffer system preparation

To give the pH = 3.5–9 buffer system (H/S buffer), histidine and succinic acid were first both dissolved together in ddH_2_O at 200 mM each. This H/S buffer stock was then split in two equal parts, which were then individually adjusted to pH 9 and 3.5 using concentrated HCl and NaOH, respectively. The obtained low-pH and high-pH H/S buffer stocks were then mixed at different ratios to give volume fractions of pH = 9 H/S buffer from 0 to 1 in 0.1 steps. These samples were then subjected to measurements using pH probes to give a calibration curve (Extended Data Fig. 1). This calibration curve was subsequently applied in future measurements to directly calculate the pH from the volume fraction of the pH = 9 H/S buffer stock. Furthermore, from the calibration curve, stocks at desired pH values could be prepared by mixing pH = 9 and pH = 3.5 H/S buffer stocks at the corresponding volume fractions. All H/S buffer stocks were stored at 4 °C.

### Fabrication of microfluidic devices

Microfluidic devices were designed using AutoCAD software (Autodesk, Version AutoCAD 2024), followed by printing on acetate transparency masks (Micro Lithography Services). The replica master was obtained via standard soft-lithography steps using spin-coating of SU-8 photoresists (MicroChem) onto polished silicon wafers^70^. Typically, SU-8 3050 was applied to achieve a device height of approximately 100 µm. After ultraviolet exposure, utilizing a custom-built LED-based apparatus^71^, the precise heights of the features were measured using a profilometer (Dektak, Bruker). Devices were then produced in polydimethylsiloxane (PDMS). PDMS (Dow Corning) was mixed in a 10:1 (w/w) ratio with a curing agent (Sylgard 184, Dow Corning) and poured onto the master, followed by degassing and baking for 1.5 h at 65 °C. The PDMS was then removed from the master and punched using biopsy punches to generate inlet holes, after which the slab was bonded onto thin glass slides using oxygen plasma surface activation (Diener electronic, 40% power for 30 s).

### Continuous pH phase boundary mapping

For the microfluidic experiment, four aqueous solutions containing peptide, ddH_2_O and both high- and low-pH H/S buffer stocks (50 mM, pH = 3.5 and 9, respectively) as well as an oil solution for droplet generation (HFE-7500 mechanical oil containing 1.2% Bio-RAN) were used. Here the peptide was tagged with Cy5-NHS ester dye (1:100 molar ratio) using a previously established procedure^72^ before application of the stock to the microfluidic chip. Both H/S stock solutions were then individually supplemented with either Alexa Fluor 488 or 647 free dye at a concentration of 5 µM, to enable tracing of the high- and low-pH buffer fraction in each droplet. The solutions were then loaded into four separate inlets on the microfluidic chip using pressure control pumps (LineUp Flow EZ, Fluigent). The aqueous solutions were first contacted before encountering the droplet junction, where droplets were formed by a constant oil flow of 100 µl h^−1^. To scan chemical phase space, the total flow rate of both H/S buffer stocks combined were kept constant while varying their flows individually between 5 µl h^−1^ and 50 µl h^−1^ and ensuring a final experiment buffer concentration of 50 mM typically. Similarly, the combined flow rate of peptide and ddH_2_O flow rate was kept constant while varying both flows individually between 5 µl h^−1^ and 50 µl h^−1^ at much shorter intervals compared with the H/S buffer flow rate variations. Droplets were subsequently incubated for 3 min on chip, followed by imaging under continuous flow using an openFrame epifluorescent microscope (Cairn Research) with a 10 × 76 air objective (Nikon CFI Plan Fluor) and equipped with a dichroic filter set (Cairn Research), allowing simultaneous imaging of three wavelengths (488 nm, 546 nm and 647 nm). Crosstalk was accounted for by imaging the stock solutions individually. These images were subsequently analysed using a custom-written Python (Python version 3.9.7) script, enabling both droplet detection and phase separation classification^73^. Phase separation classification was assigned as mixed = 0 and phase separated = 1. The fraction of phase-separated conditions is then calculated by application of binning based on established literature algorithms^74,75^ (Extended Data Fig. 2). To calibrate, microdrops filled with only one stock solution at a time are generated and subsequently images in the corresponding fluorescent channel. This allows for correcting the illumination profile by dividing by the average intensity of many frames and then fit a Gaussian to the intensity histogram of the calibration images to obtain an intensity value that corresponds to the stock solution’s concentration (Extended Data Fig. 2). The same process is applied for a background image (droplets with no corresponding dye), and a calibration curve of droplet intensity to concentration is generated. The process is repeated for all fluorescent solutions. For mapping intensity to the pH of the droplets, normalized intensities in the respective high- and low-pH fluorescent wavelengths were used to establish the volume fractions of high- and low-pH buffers.

### Sample preparation and phase separation conditions

Phase separation was exclusively induced in vitro by gently mixing components in 500 µl Eppendorf tubes, where the specific conditions investigated are reported for each specific dataset. To probe phase separation of FUS under varying concentrations of TRIS, ddH_2_O was first added to the Eppendorf tube, followed by addition of KCl (1 M in ddH_2_O) to give a constant final concentration of 150 mM considering an addition of TRIS (1 M, pH = 7.3) to obtain a range of 20–200 mM final concentration. Lastly, the protein (70 µM, 750 mM KCl, 50 mM TRIS buffer pH = 7.3) was added to yield a final concentration of 1 µM at a total volume of 10 µl. Fluorescence was then observed via an inverted microscope (openFrame, Cairn Research) set up with a 20× air objective (Nikon) combined with appropriate filters (Laser2000). Imaging was then performed with a high-sensitivity camera (Prime BSI Express sCMOS, Photometrics).

Insulin dilute-phase concentration measurements under varying buffer concentration conditions were performed by first diluting high- and low-pH H/S buffer (200 mM, pH = 9 and pH = 3.5, respectively) individually to the desired concentration such as 1/10 in ddH_2_O for a final concentration of 20 mM. Insulin stocks were then prepared at 5 mg ml^−1^ in high-pH H/S buffer at the same buffer concentration. Samples were then prepared by mixing the insulin stock with high- and low-pH H/S buffer at 120 µl, 105 µl and 75 µl each to give a final ratio of 1/3 of low- to high-pH H/S buffer with a pH of 6.4 (see Extended Data Fig. 1 for H/S buffer pH calibration) and 300 µl total volume at 2 mg ml^−1^ final insulin concentration. Samples at varying buffer concentrations were then simply generated in the same way by mixing insulin and H/S buffer stocks with the desired buffer concentration. Experiments at a final insulin concentration of 1 mg ml^−1^ were prepared in the same way using 2.5 mg ml^−1^ insulin stocks. Insulin dilute-phase concentration measurements under varying pH conditions were performed by mixing insulin stock (10 mg ml^−1^, 100 mM H/S buffer, pH = 9) at a 1/5 ratio with high- and low-pH H/S buffer stocks (100 mM, pH = 9 and pH = 3.5, respectively) combined to yield the desired pH. The samples were then subsequently centrifuged for 10 min, followed by decanting of the supernatant and measurement of the intrinsic supernatant absorbance at 280 nm.

### SNARF-4F pH calibration and readouts

SNARF-4F calibration samples were prepared by mixing high- and low-pH H/S buffer (100 mM) at varying volume fractions to give stocks in the pH range between 3.5 and 9, followed by addition of SNARF-4F (in DMSO, 10 mM) to a final concentration of 20 µM. These samples were then first used to characterize the changes in the SNARF-4F emission spectra using a Cary Eclipse fluorescence spectrophotometer (Varian) with an excitation wavelength (*λ*) of 488 nm and scanning emission (Em) between 520 nm and 730 nm with a rate of 120 nm min^−1^ and collecting intensity every nanometre. The excitation slit used was 5 nm and emission slit 10 nm. Then, to establish the confocal imaging approach, 10 µl of the SNARF-4F calibration samples was deposited on clean 24 × 50 mm no. 1 cover glass slides (VWR) and imaged via a Leica Stellaris 5 confocal microscope equipped with a 63× oil immersion objective (Leica HC PL APO 63×/1.40 Oil CS2, numerical aperture 1.4). Here high-resolution images were taken (2,048 × 2,048 pixels) by excitation at 488 nm and acquiring at 580 ± 10 nm and 650 ± 10 nm simultaneously. The laser power was kept at 2%, and the detector gain was set to 60%. The images obtained at extreme pH conditions (pH = 3.5 and 9) were then simply used to set up the pH reference calibration according to the supplier manual, where Em_1_ = *λ*_1_ = 580 ± 10 nm and Em_2_ = *λ*_2_ = 650 ± 10 nm. Phase-separated samples for dense- and dilute-phase pH measurements using SNARF-4F were acquired in the same way and prepared by mixing components in 500 µl Eppendorf tubes. The specific conditions investigated are reported for each specific dataset, and SNARF-4F was consistently applied at a final concentration of 20 µM. Analysis of the dense- and dilute-phase pH was then performed by feature segmentation of dense-phase compartments into image masks using the ilastik segmentation toolkit^76,77^ (Extended Data Fig. 5). The dilute-phase Em_1_ and Em_2_ intensity was then calculated as the average non-dense-phase intensity. The Em_1_ and Em_2_ intensity of each individual dense-phase compartment was calculated by averaging over all pixels associated to only that specific dense-phase compartment.

### Hydrodynamic radius determinations using microfluidic diffusional sizing

Microfluidic diffusional sizing was performed using the Fluidity One-M instrument (Fluidic Analytics) based on a previously published operation principle^78^. In brief, 4 µl of sample at 100 nM protein concentration (unless specified otherwise) was added to the chip after the auxiliary channels were primed with buffer (pH = 7.3, 50 mM TRIS buffer, 150 mM KCl). Samples were then measured in triplicates based on detection in the Alexa488 (PGL3-GFP, pK-FITC).

### Multiphasic condensate imaging

Multiphasic PGL3, pK and p(A)-RNA condensates were formed by mixing 5 µM of PGL3-EGFP, 5 µM of pK tagged with Alexa647 and 10 ng µl^−1^ RNA in an Eppendorf tube at a total volume of 20 µl and buffer conditions of 150 mM KCl and 50 mM TRIS. The pK labelling was performed according to a previously established NHS-ester covalent conjugation protocol^72^. Samples were then imaged after 5 min of incubation by deposition on clean 24 × 50 mm no. 1 cover glass slides (VWR) and imaged via a Leica Stellaris 5 confocal microscope equipped with a 63× oil immersion objective (Leica HC PL APO 63×/1.40 Oil CS2, NA 1.4), taking high-resolution images (2,048 × 2,048 pixels).

### Insulin dilute-phase concentration measurements

Insulin phase-separating samples were prepared at specified conditions, for example, 2 mg ml^−1^ insulin in 50 mM H/S buffer at pH = 6.4, by addition of insulin from a dissolved pH = 9.0 H/S buffer stock at a total of 1 ml, followed by incubation over 10 min. Subsequently, samples were centrifuged for 5 min, after which the supernatant was decanted and dilute-phase concentrations were determined using 280 nm absorbance measurements.

### FUS dilute-phase concentration measurements

Given the low material volumes available, FUS dilute-phase concentrations were performed by a previously published flow-cell coupled scanning confocal approach^18,33^. In brief, FUS samples were first prepared at specified conditions in Eppendorf tubes at a total volume of 10 µl, and following 30 min incubation, microfluidic chips were flushed with the sample by applying negative pressure. Sample time traces were then recorded using a home-built confocal set-up equipped with picosecond-pulsed 485 nm laser sources as well as a 60× water-immersion objective (CFI Plan Apochromat WI 60×, NA 1.2, Nikon). The dilute-phase concentration was then extracted from the time trace as the baseline signal given the large excess of the dilute-phase volume fraction compared with the dense phase.

### Sequence-based physico-chemical property calculations

Physico-chemical properties of individual protein sequences were calculated directly from the sequence using tabled properties of the individual amino acids by applying the Bio.SeqUtils.ProtParam Python package functions .charge_at_pH() and .isoelectric_point(). The electrochemical reconstitution of complex mixtures of sequences was performed by first calculating the charge-dependent pH profiles of the individual sequences, followed by summation and normalization for the total number of protein sequences. Here the mixture pI was simply determined as the point where this effective mixture charge profile reaches zero net charge. The sequence charge density *q* was determined by normalizing the net charge at a given pH with the number of residues. The sequence hydrophobicity was evaluated using Kyte–Doolittle scaling. Species proteomes were downloaded from UniProt. pH-dependent RNA charge calculation was performed using a simplified, site-based approach where each nucleotide in the RNA chain is treated as a combination of a nucleobase and a phosphate group. Each nucleotide is considered as comprised of a nucleobase and a phosphate group. For nucleobases (A, C, G and U), approximate p*K*_a_ values are assigned, and the fraction of protonation was determined via the Henderson–Hasselbalch equation, thereby estimating any positive charge contributions. Simultaneously, each phosphate group is modelled with two sequential deprotonation steps—each with its own p*K*_a_—resulting in net negative charge as deprotonation increases. Finally, the contributions from all nucleobases and phosphate groups across the RNA sequence are summed to produce a theoretical net charge at a given pH.

### Evaluation of impact of folding on predicted pI using PypKA

To consider folding-induced shifts on the calculated protein pI, PypKA was applied^40^, which takes as input a three-dimensional PDB structure file and uses a mixed Poisson–Boltzmann and Monte Carlo approach to attain a pI accounting for the protein’s folded state. Application to FUS and PGL3 (Extended Data Fig. 7a) showed limited differences between sequence-based and structure-corrected pI, indicating that in these cases simplified sequence analysis is sufficient to capture the electrostatic properties of these proteins. For folding effect testing at scale, a similar comparison was performed for a subset of 575 proteins obtained by filtering for SwissProt/UniProt Human proteins and focusing on proteins where full-length PDB chains were available (Extended Data Fig. 7). No statistical significance between the two methods (Wilcoxon signed-rank test, *P* = 0.14) was observed, while only 15% of proteins have an absolute error greater than 1.5 pI units when not accounting for the protein’s folded state.

### Generation of randomized proteomes

To obtain information of the physico-chemical property distributions of randomized proteomes, proteins were generated by randomly concatenating individual amino acids at given probability of occurrence for the individual amino acids and total protein length. For the set of fully randomized sequences, an even probability of occurrence was assumed for the individual amino acids and proteins were constrained to 560 amino acids in total length, corresponding to the average protein length in the human proteome. To obtain a ‘humanized’ random proteome, the probability of occurrence for the individual amino acids was amended to be the same as that of the human proteome and similarly sequences were generated at lengths corresponding to the human proteome length distribution. In each case, a total of 500,000 sequences were generated and subsequently evaluated for their physico-chemical properties.

### PhaseSepDB condensate proteomes

PhaseSepDB v2 data were loaded and processed in Python where only ‘Homosapien’ entries were used^44^. For each membraneless organelle (MLO) in the database, there was a record of which proteins had this MLO annotation. Taking the MLOs with nine most common annotations, the physico-chemical properties of the MLO proteome were evaluated as outlined above. MLOs with fewer annotations were deemed potentially unreliable to apply this method.

### Condensate Atlas usage

Protein clusters from the Condensate Atlas were used if more than 50% of the involved proteins were deemed a ‘scaffold’ (high homotypic phase separation prediction score) as outlined in ref. ^48^. Then, physico-chemical parameters such as charge and mixed pI of the condensates were calculated as outlined above.

## Online content

Any methods, additional references, Nature Portfolio reporting summaries, source data, extended data, supplementary information, acknowledgements, peer review information; details of author contributions and competing interests; and statements of data and code availability are available at 10.1038/s41557-025-02039-9.

## Supplementary information

Supplementary Information Supplementary Notes and references.

Supplementary Data 1 Description of pH model.

Supplementary Code 2 Code repository.

## Source data

Source Data Figs. 1–6 Microfluidic phase boundary mapping data, dilute phase concentration measurements, condensate SNARF dye readout data, sequence charge data, dominance analysis data, spatial profile SNARF dye readout data, microfluidic diffusional sizing data, confocal imaging data, protein pI data, proteome wide analysis data, Condensate Atlas data and theoretical model data.

Source Data Extended Data Figs. 1–10 pH readout data, microfluidic phase boundary mapping data, dilute phase concentration measurements, dominance analysis data, sequence charge data, condensate SNARF dye readout data, fluorimeter data, spatial profile SNARF dye readout data, PypKA data, proteome wide analysis data, protein pI data and Condensate Atlas data.

## Data Availability

Source data are provided with this paper.

## Code availability

Analysis code is provided with the paper where applicable.
