# Supplementary material for: Biomolecular condensates sustain pH gradients at equilibrium through charge neutralization
Source: Nat Chem. 2026 Jan 29;18(2):246–57. doi: 10.1038/s41557-025-02039-9 (PMC12872462; doi:10.1038/s41557-025-02039-9)
Supplement: Supplementary file 1 — Supplementary Notes and references. [file 41557_2025_2039_MOESM1_ESM.pdf]

nature chemistry

Supplementary information https://doi.org/10.1038/s41557-025-02039-9

Biomolecular condensates sustain pH gradients at equilibrium through charge neutralization

In the format provided by the authors and unedited

Table of Contents

- **Supplementary Notes**
- **References**

Supplementary Notes

Small molecule buffer partitioning

To quantify the potential origin of the dense phase buffering effect, we measured the insulin c_{dil} at varying concentrations of H/S buffer but fixed pH = 6.4 and total peptide concentration (Extended Data Fig. 3). Here, c_{dil} decreases with decreasing H/S buffer concentration highlighting a competition between the environmental buffer strength and phase separation. This can be explained as the dense phase has to act as a distinct buffering system against the environmental pH to generate the pH gradient necessary to minimise repulsion. By combining data sets at 2 and 1.5 mg/mL total insulin concentration, we can then also perform partitioning analysis for the H/S buffer[1,2]. This yields a negative reduced tie-line gradient, i.e. that the buffer concentration is lower in the dense phase than the dilute phase. Hence, regulation of the dense phase pH is aided by buffer exclusion to favour the buffering of protein associated side groups.

Reduced tie line approach in pH chemical space

To interpret the dilute phase band gradients obtained via the microdroplet pH scanning approach, we first remark that the construction of these bands depends on mass balance, and in the present case the conservation is to be applied to the difference in total concentrations of H+ ions and OH- ions, regardless of whether they are in the free solution state or bound to the buffer, or each other. Mass conservation works for this difference because dissociation of them from buffer molecules does not affect their total concentrations, and dissociation of water releases equal amounts of both to the solution so the difference does not change. The dilute phase bands then indicate how this differential concentration, i.e. the difference between total $[H^+]$ and total $[OH^-]$, is partitioning across phases. To make the connection between the differential concentration and pH, we note that there exists a highly non-linear but monotonic mapping between the two, so each pH on the x-axis of the pH phase boundary corresponds to a particular value of the partial H^+/OH^- total concentration difference, with higher pH corresponding more OH^- compared to H^+. A positive dilute phase band gradient at the low-pH section then means the dense phase total $[OH^-]$ **-** $[H^+]$ is higher than the dilute phase, translating to a higher pH in the dense phase and vice versa for the high-pH section.

References

1. Qian, D. *et al.* Tie-Line Analysis Reveals Interactions Driving Heteromolecular Condensate Formation. *Phys. Rev. X* **12**, 041038 (2022).

2. Ausserwöger, H. *et al.* Quantifying collective interactions in biomolecular phase separation. *Nature Communications* **16**, 7724 (2025).
