# Supplementary material for: Biomolecular condensates sustain pH gradients at equilibrium through charge neutralization
Source: Nat Chem. 2026 Jan 29;18(2):246–57. doi: 10.1038/s41557-025-02039-9 (PMC12872462; doi:10.1038/s41557-025-02039-9)
Supplement: Supplementary file 2 — Description of pH model. [file 41557_2025_2039_MOESM2_ESM.pdf]

pH-Model

I. 1 DERIVING THE PH OF WATER

We now consider the water autodissociation reaction written as

$$2\,H_2O \rightleftharpoons H_3O^+ + OH^-,$$

which describes the transfer of a proton from one water molecule to another, forming a hydronium ion.

We use the conservation of oxygen and hydrogen to obtain

$$c_{H_2O} + c_{H_3O} + c_{OH} = W. \tag{1}$$

Charge neutrality enforces

$$c_{H_3O} = c_{OH} = c \tag{2}$$

And combining the above two equations gives

$$c_{H_2O} = W - 2c \tag{3}$$

Free Energy of Dissociation

The free energy change associated with the formation of the hydronium–hydroxide pair is

$$\Delta G = \sum_j \nu_j c_j \mu_j + c\Delta_{\rm dis}G, \tag{4}$$

where ΔG_{dis} represents the energetic term of the dissociation reaction corresponding to bond breaking and forming.

$$\Delta G = c\,\mu_{H_3O^+} + c\,\mu_{OH^-} - 2(W-2c)\mu^\circ_{H_2O} + c\,\Delta_{\rm dis}G, \tag{5}$$

and c denotes the concentration of both H_3O^+ and OH^-.

Chemical Potentials

Assuming ideal behavior, the chemical potential of species i is given by

$$\mu_i = \mu_i^\circ + RT\ln\frac{c_i}{c^\circ}, \tag{6}$$

with the standard concentration $c^\circ = 1\,\mathrm{M}$. Hence,

$$\mu_{H_3O^+} = \mu^\circ_{H_3O^+} + RT\ln c, \quad \mu_{OH^-} = \mu^\circ_{OH^-} + RT\ln c.$$

Substituting these into the expression for ΔG yields

$$\Delta G = c\mu^\circ_{H_3O^+} + c\mu^\circ_{OH^-} - 2(W-2c)\mu^\circ_{H_2O} + c\Delta_{\rm dis}G + c\,RT\ln\frac{c^2}{c^{\circ 2}} \tag{7}$$

or

$$\Delta G = \Delta G^\circ + c\Delta_{\rm dis}G + c\,RT\ln\frac{c^2}{c^{\circ 2}} \tag{8}$$

Equilibrium Condition

At equilibrium, ΔG is minimized with respect to c. Differentiating with respect to c and setting the derivative to zero gives

$$\frac{d\Delta G}{dc} = \Delta_{\text{dis}} G + 2\,RT\Big(1 + \ln\frac{c}{c^{\circ}}\Big) = 0. \tag{9}$$

Expressing the dissociation free energy in terms of the equilibrium constant K_w by

$$\Delta_{\text{dis}} G = -RT \ln K_w.$$

gives:

$$c = c^{\circ} \exp\Big[\frac{1}{2}\Big(\ln K_w - 2\Big)\Big]. \tag{10}$$

Which gives roughly a pH of 7 when suitable equilibrium constants are chosen (though we note a discrepancy in equilibrium constants may shift this slightly [1])

II. CONCISE DERIVATION

Now we continue with a concise derivation, dropping chemical potential conventions and standard concentrations and work solely in concentrations. At the end, we review the relationship between the two scenarios before adding extra components to our system

For pure water, the dissociation reaction conserves the difference between H and OH groups

$$c_{\mathrm{H_3O}} = c_{\mathrm{OH}}, \tag{11}$$

and mass conservation of oxygen requires, for a constant W,

$$c_{\mathrm{H_2O}} + c_{\mathrm{H_3O}} + c_{\mathrm{OH}} = W. \tag{12}$$

It is easy to check that

$$2c_{\mathrm{H_2O}} + 3c_{\mathrm{H_3O}} + c_{\mathrm{OH}} = 2W, \tag{13}$$

as required by conservation of hydrogen. The free proton concentration $c_{\mathrm{H_3O}}$ is a non-conserved order parameter. The dissociation introduces an energy change, and the total free energy of the system can be written as, when the concentrations are measured in a reference concentration,

$$f = c_{\mathrm{H_2O}} \ln c_{\mathrm{H_2O}} + c_{\mathrm{H_3O}} \ln c_{\mathrm{H_3O}} + c_{\mathrm{OH}} \ln c_{\mathrm{OH}} - \Delta_1 c_{\mathrm{H_2O}} = (W - 2c_{\mathrm{H_3O}}) \ln(W - 2c_{\mathrm{H_3O}}) + 2c_{\mathrm{H_3O}} \ln c_{\mathrm{H_3O}} - \Delta_1 (W - 2c_{\mathrm{H_3O}}). \tag{14}$$

The free energy minimum can be calculated now

$$\frac{\mathrm{d}f}{\mathrm{d}c_{\mathrm{H_3O}}} = (2\ln c_{\mathrm{H_3O}} + 2) - [2\ln(W - 2c_{\mathrm{H_3O}}) + 2] + 2\Delta_1 = 2\ln\frac{c_{\mathrm{H_3O}}}{W - 2c_{\mathrm{H_3O}}} + 2\Delta_1 \approx 2\ln\frac{c_{\mathrm{H_3O}}}{W} + 2\Delta_1, \tag{15}$$

and setting it to 0 in principle gives pH of 7 when a realistic value of Δ_1 is used:

$$c_{\mathrm{H_3O}} = W e^{-\Delta_1}. \tag{16}$$

Comparing this to equation 10, we note that W is the standard concentration for our system, and Δ_1 is a function of the dissociation equilibrium constant

III. HCL (BUFFER)

Assume we now add a certain amount B of HCl (or any salt buffer) to the solution. Since the solution is charge neutral, we have

$$c_{\mathrm{H_3O}} - c_{\mathrm{OH}} - c_{\mathrm{Cl}} = 0, \tag{17}$$

where the c_{Cl} was missing from before. This allows us to substitute c_{OH} with

$$c_{\mathrm{OH}} = c_{\mathrm{H_3O}} - c_{\mathrm{Cl}}. \tag{18}$$

Mass conservation of chloride gives

$$c_{\mathrm{HCl}} + c_{\mathrm{Cl}} = B, \tag{19}$$

and for water (oxygen)

$$c_{\mathrm{H_2O}} + c_{\mathrm{H_3O}} + c_{\mathrm{OH}} = c_{\mathrm{H_2O}} + 2c_{\mathrm{H_3O}} - c_{\mathrm{Cl}} = W. \tag{20}$$

The amount of dissociated HCl is c_{Cl}, so the total free energy can be written as

$$\begin{aligned} f &= c_{\mathrm{H_2O}} \ln c_{\mathrm{H_2O}} + c_{\mathrm{H_3O}} \ln c_{\mathrm{H_3O}} + c_{\mathrm{OH}} \ln c_{\mathrm{OH}} \\ &\quad + c_{\mathrm{Cl}} \ln c_{\mathrm{Cl}} + c_{\mathrm{HCl}} \ln c_{\mathrm{HCl}} \\ &\quad - \Delta_1 c_{\mathrm{H_2O}} - \Delta_2 c_{\mathrm{HCl}} \\ &= (W - 2c_{\mathrm{H_3O}} + c_{\mathrm{Cl}}) \ln(W - 2c_{\mathrm{H_3O}} + c_{\mathrm{Cl}}) + c_{\mathrm{H_3O}} \ln c_{\mathrm{H_3O}} + (c_{\mathrm{H_3O}} - c_{\mathrm{Cl}}) \ln(c_{\mathrm{H_3O}} - c_{\mathrm{Cl}}) \\ &\quad + c_{\mathrm{Cl}} \ln c_{\mathrm{Cl}} + (B - c_{\mathrm{Cl}}) \ln(B - c_{\mathrm{Cl}}) \\ &\quad - \Delta_1(W - 2c_{\mathrm{H_3O}} + c_{\mathrm{Cl}}) - \Delta_2(B - c_{\mathrm{Cl}}), \end{aligned} \tag{21}$$

which is a function of both $c_{\mathrm{H_3O}}$ and c_{Cl}. Now $f = f(c_{\mathrm{H_3O}}, c_{\mathrm{Cl}})$ has to be minimised with respect to the amount of HCl and water dissociated, meaning we need to solve the simultaneous equations

$$\frac{\partial f}{\partial c_{\mathrm{H_3O}}} = 0, \tag{22a}$$

$$\frac{\partial f}{\partial c_{\mathrm{Cl}}} = 0. \tag{22b}$$

$$\tag{22c}$$

The first equation gives

$$c_{\mathrm{H_3O}}(c_{\mathrm{H_3O}} - c_{\mathrm{Cl}}) = (W - 2c_{\mathrm{H_3O}} + c_{\mathrm{Cl}})^2 e^{-2\Delta_1}, \tag{23}$$

which is effectively, assuming

$$W - 2c_{\mathrm{H_3O}} + c_{\mathrm{Cl}} \approx W, \tag{24}$$

$$c_{\mathrm{H_3O}} c_{\mathrm{OH}} = W^2 e^{-2\Delta_1}, \tag{25}$$

and the second

$$\frac{c_{\mathrm{Cl}}}{(c_{\mathrm{H_3O}} - c_{\mathrm{Cl}})(B - c_{\mathrm{Cl}})} = \frac{1}{W} e^{-\Delta_2 + \Delta_1}, \tag{26}$$

which can be re-written as

$$\begin{aligned} \frac{c_{\mathrm{Cl}}}{(c_{\mathrm{H_3O}} - c_{\mathrm{Cl}})(B - c_{\mathrm{Cl}})} &= \frac{1}{W} e^{-\Delta_2 + \Delta_1} \\ \frac{c_{\mathrm{Cl}}}{c_{\mathrm{OH}} c_{\mathrm{HCl}}} &= \frac{1}{W} e^{-\Delta_2 + \Delta_1} \\ \frac{c_{\mathrm{Cl}} c_{\mathrm{H_3O}}}{c_{\mathrm{HCl}}} &= W e^{-\Delta_2 - \Delta_1}, \end{aligned} \tag{27}$$

as we would expect. In principle, we can now solve for the free energy minimiser concentrations, denoted by $c^*_{\mathrm{H_3O}}$ and c^*_{Cl}. Once the minimisation is done, we obtain the free energy of the solution at equilibrium f^* as

$$f^* = f[c^*_{\mathrm{H_3O}}(B), c^*_{\mathrm{Cl}}(B)], \tag{28}$$

where the dependence of B is written out explicitly. Conversely, we can view the three variables $c^*_{\mathrm{H_3O}}$, c^*_{Cl}, and B as a set of unknowns constrained by the two equations from free energy minimisation, leaving exactly one degree of freedom. This means we are effectively free to specify one of $\{c^*_{\mathrm{H_3O}}, c^*_{\mathrm{Cl}}, B\}$, and the whole state is set.

IV. POLYMER WITH PI

Suppose the neutral polymer species is PH, with two different pKa values for a cationic protonated state HPH and an anionic de-protonated state P. The total polymer amount is C. Then

$$c_{\mathrm{H_3O}} + c_{\mathrm{HPH}} - c_{\mathrm{OH}} - c_{\mathrm{P}} = 0, \tag{29a}$$
$$c_{\mathrm{HPH}} + c_{\mathrm{PH}} + c_{\mathrm{P}} = C, \tag{29b}$$
$$c_{\mathrm{H_2O}} + c_{\mathrm{H_3O}} + c_{\mathrm{OH}} = W. \tag{29c}$$

These correspond to charge neutrality, polymer mass conservation, and oxygen mass conservation. Make the substitutions

$$c_{\mathrm{OH}} = c_{\mathrm{H_3O}} + c_{\mathrm{HPH}} - c_{\mathrm{P}}, \tag{30a}$$
$$c_{\mathrm{HPH}} = C - c_{\mathrm{PH}} - c_{\mathrm{P}}, \tag{30b}$$
$$c_{\mathrm{H_2O}} = W - c_{\mathrm{H_3O}} - c_{\mathrm{OH}} = W - 2c_{\mathrm{H_3O}} - c_{\mathrm{HPH}} + c_{\mathrm{P}}. \tag{30c}$$

The overall system free energy is

$$\begin{aligned} f = {} & c_{\mathrm{H_2O}} \ln c_{\mathrm{H_2O}} + c_{\mathrm{H_3O}} \ln c_{\mathrm{H_3O}} + c_{\mathrm{OH}} \ln c_{\mathrm{OH}} \\ & + c_{\mathrm{P}} \ln c_{\mathrm{P}} + c_{\mathrm{PH}} \ln c_{\mathrm{PH}} + c_{\mathrm{HPH}} \ln c_{\mathrm{HPH}} \\ & - \Delta_1 c_{\mathrm{H_2O}} - \Delta_3 c_{\mathrm{HPH}} - \Delta_4 c_{\mathrm{P}} \\ & + \chi_q (c_{\mathrm{HPH}} - c_{\mathrm{P}})^2 + \chi_f C^2. \end{aligned} \tag{31}$$

The equilibration equations now involve more species, and collectively they give

$$c_{\mathrm{H_3O}} c_{\mathrm{OH}} = W^2 e^{-2\Delta_1}, \tag{32a}$$
$$\frac{c_{\mathrm{P}} c_{\mathrm{H_3O}}}{c_{\mathrm{PH}}} = W e^{-(\Delta_1 - \Delta_4) - 2(c_{\mathrm{P}} - c_{\mathrm{HPH}})\chi_q}, \tag{32b}$$
$$\frac{c_{\mathrm{PH}} c_{\mathrm{H_3O}}}{c_{\mathrm{HPH}}} = W e^{-(\Delta_1 + \Delta_3) - 2(c_{\mathrm{P}} - c_{\mathrm{HPH}})\chi_q}. \tag{32c}$$

$(\Delta_1 - \Delta_4)$ plays the role of the pKa value for the acidic group, and $(\Delta_1 + \Delta_3)$ corresponds to the pKa of the basic group.

V. BUFFERED POLYMER SOLUTION

Now consider a solution with both salt and polymer present. The conservation equations are

$$c_{\mathrm{H_3O}} + c_{\mathrm{HPH}} - c_{\mathrm{OH}} - c_{\mathrm{P}} - c_{\mathrm{Cl}} = 0, \tag{33a}$$
$$c_{\mathrm{HCl}} + c_{\mathrm{Cl}} = B, \tag{33b}$$
$$c_{\mathrm{HPH}} + c_{\mathrm{PH}} + c_{\mathrm{P}} = C, \tag{33c}$$
$$c_{\mathrm{H_2O}} + c_{\mathrm{H_3O}} + c_{\mathrm{OH}} = W. \tag{33d}$$

The free energy is now

$$
\begin{aligned}
f = {} & c_{\mathrm{H_2O}} \ln c_{\mathrm{H_2O}} + c_{\mathrm{H_3O}} \ln c_{\mathrm{H_3O}} + c_{\mathrm{OH}} \ln c_{\mathrm{OH}} \\
& + c_{\mathrm{Cl}} \ln c_{\mathrm{Cl}} + c_{\mathrm{HCl}} \ln c_{\mathrm{HCl}} \\
& + c_{\mathrm{P}} \ln c_{\mathrm{P}} + c_{\mathrm{PH}} \ln c_{\mathrm{PH}} + c_{\mathrm{HPH}} \ln c_{\mathrm{HPH}} \\
& - \Delta_1 c_{\mathrm{H_2O}} - \Delta_2 c_{\mathrm{HCl}} - \Delta_3 c_{\mathrm{HPH}} - \Delta_4 c_{\mathrm{P}} \\
& + \chi_q (c_{\mathrm{HPH}} - c_{\mathrm{P}})^2 + \chi_f C^2 .
\end{aligned} \tag{34}
$$

Minimising with respect to the various species gives

$$c_{\mathrm{H_3O}} c_{\mathrm{OH}} = W^2 e^{-2\Delta_1}, \tag{35a}$$

$$\frac{c_{\mathrm{P}} c_{\mathrm{H_3O}}}{c_{\mathrm{PH}}} = W e^{-(\Delta_1 - \Delta_4) - 2(c_{\mathrm{P}} - c_{\mathrm{HPH}})\chi_q}, \tag{35b}$$

$$\frac{c_{\mathrm{PH}} c_{\mathrm{H_3O}}}{c_{\mathrm{HPH}}} = W e^{-(\Delta_1 + \Delta_3) - 2(c_{\mathrm{P}} - c_{\mathrm{HPH}})\chi_q}, \tag{35c}$$

$$\frac{c_{\mathrm{Cl}} c_{\mathrm{H_3O}}}{c_{\mathrm{HCl}}} = W e^{-(\Delta_1 + \Delta_2)}. \tag{35d}$$

These relations can be written down directly from high school chemistry, but the point is that we need to calculate f and deduce the stability of the solution from there.

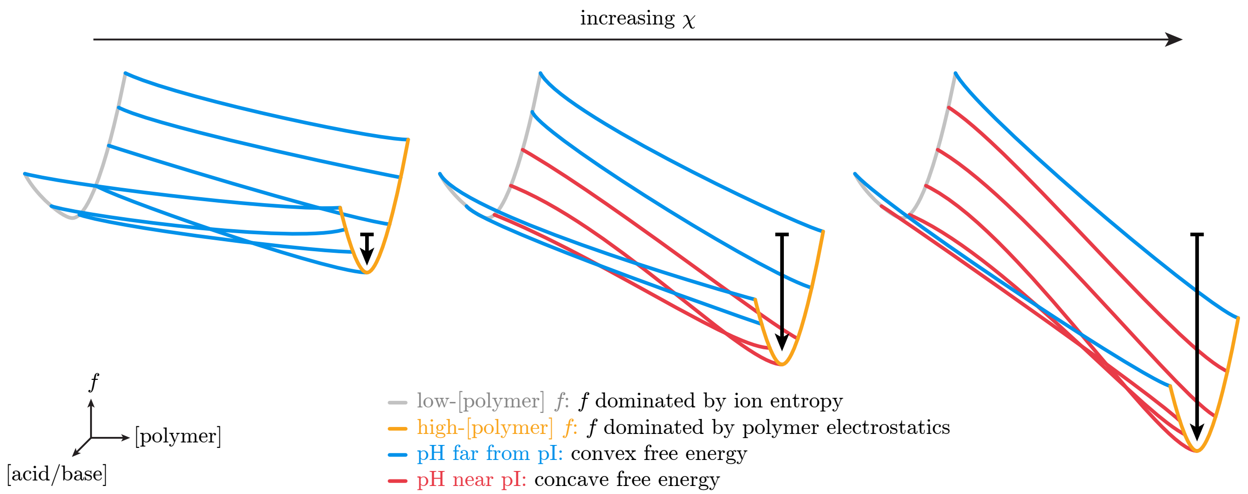

FIG. 1. For a pure buffer solution, the total system free energy (grey) is increasingly convex at high or low total pH, with the total pH set via addition of acids or bases. At high polymer concentration (orange), the convexity comes from electrostatic repulsion, and the free energy attains a minimum near the polymer iso-electric point. The full free energy bridges these two limits, and phase separation may or may not occur depending on the convexity of this surface. The polymer self-attraction χ introduces a concave term of the form $-\chi C^2$ with C the total polymer concentration. When χ is weak (left), the full free energy is convex and no phase separation happens. At intermediate χ (middle) the free energy becomes concave at pH values close to the polymer's pI. At large χ (right) more parts of the free energy surface becomes concave.

[1] T. P. Silverstein and S. T. Heller, "pka values in the undergraduate curriculum: What is the real pka of water?," *Journal of Chemical Education*, vol. 94, no. 6, pp. 690–695, 2017.
